# Analysis of *NIA* and *GSNOR* family genes and nitric oxide homeostasis in response to wheat-leaf rust interaction

**DOI:** 10.1038/s41598-021-04696-5

**Published:** 2022-01-17

**Authors:** Deepak T. Hurali, Ramesh Bhurta, Sandhya Tyagi, Lekshmy Sathee, Adavi B. Sandeep, Dalveer Singh, Niharika Mallick, Vinod, Shailendra K. Jha

**Affiliations:** 1 Division of Genetics, ICAR-Indian Agricultural Research Institute, New Delhi, 110012 India; 2 Division of Plant Physiology, ICAR-Indian Agricultural Research Institute, New Delhi, 110012 India

**Keywords:** Biotic, Plant immunity, Plant molecular biology, Plant physiology, Plant signalling

## Abstract

Nitric oxide (NO) modulates plant response to biotic and abiotic stresses by S-nitrosylation-mediated protein post-translational modification. Nitrate reductase (NR) and S-nitrosoglutathione reductase (GSNOR) enzymes are essential for NO synthesis and the maintenance of Nitric oxide/S-nitroso glutathione (NO/GSNO) homeostasis, respectively. S-nitrosoglutathione, formed by the S-nitrosylation reaction of NO with glutathione, plays a significant physiological role as the mobile reservoir of NO. The genome-wide analysis identified nine *NR* *(NIA)* and three *GSNOR* genes in the wheat genome. Phylogenic analysis revealed that the nine *NIA* genes +were clustered into four groups and the 3 *GSNOR*s into two groups. qRT-PCR expression profiling of *NIAs* and GSNORs was done in Chinese spring (CS), a leaf rust susceptible wheat line showing compatible interaction, and Transfer (TR), leaf rust-resistant wheat line showing incompatible interaction, post-inoculation with leaf rust pathotype 77–5 (121-R-63). All the *NIA* genes showed upregulation during incompatible interaction in comparison with the compatible reaction. The *GSNOR* genes showed a variable pattern of expression: the *TaGSNOR1* showed little change, whereas *TaGSNOR2* showed higher expression during the incompatible response. *TaGSNOR3* showed a rise of expression both in compatible and incompatible reactions. Before inoculation and after 72 h of pathogen inoculation, NO localization was studied in both compatible and incompatible reactions. The S-nitrosothiol accumulation, NR, and glutathione reductase activity showed a consistent increase in the incompatible interactions. The results demonstrate that both NR and GSNOR plays significant role in defence against the leaf rust pathogen in wheat by modulating NO homeostasis or signalling.

## Introduction

Among the various biotic stresses, rusts are highly devastating and is a challenging pathogen to combat. The aerial spread, production of uredospores in large quantity, and rapid evolution of new pathotypes within short time intervals makes it a devastative pathogen^1^. In the case of wheat *(Triticum aestivum* L.), three rusts (stem rust, leaf rust, stripe rust) caused by the *Puccinia* species results in severe damage and yield reduction. Out of the three rusts, leaf rust, caused by *P. triticina* Eriks., is the most prevalent in major part of wheat-growing areas worldwide^2–4^. To cope with leaf rust pathogen and to improve leaf rust resistance in wheat, 80 leaf rust (LR) resistance genes have been designated^4–6^ and are being used in breeding programs. In the recent past, the cloning of resistance genes have improved the understanding of leaf rust resistance in wheat albeit, the signalling mechanism behind resistance is not fully understood^7^.

Pathogen infection elicits pathogen-associated molecular patterns (PAMP) triggered immunity (PTI) as the first line of defence in plants. Effector triggered immunity (ETI) is the second line of defence after the breakdown of PTI. In the case of ETI, the effector molecules released by the pathogen are recognized directly or indirectly by the *R* gene product and results in localized cell death known as the hypersensitive response (HR)^7^. Many signalling molecules are involved in triggering HR reactions in plants. Among them, reactive oxygen species (ROS), reactive nitrogen species (RNS), Salicylic acid (SA), and Jasmonic acid (JA) are the main players^8^. In recent years, Nitric oxide (NO) has gained importance as a central signalling molecule in animals and plants^9^. Nitric oxide, also called nitrogen monoxide (IUPAC), is a colourless gaseous free radical found ubiquitously in most living organisms^10^. Nitric oxide is a highly diffusible and reactive gaseous radical endogenously produced by plants. Nitric oxide is the first gasotransmitter molecule identified, which can affect the function of cells by modifying the target proteins^11^.

Nitric oxide plays an essential role in plant immunity by activating the pathogenesis-related proteins in plants^12^. Balanced production of NO and ROS intermediates are required for hypersensitive cell death^13^. In plants, two major pathways of NO synthesis are known, an oxidative pathway similar to the animal NO synthase (NOS) pathway and the reductive pathway. NOS genes are yet to be identified and characterized in the plant kingdom. The reductive way is catalyzed by nitrate reductase (NR), a cytosolic enzyme^14,15^. The *Arabidopsis* NR1 and NR2 (*AtNIA1*/*AtNIA*2) are associated with NO synthesis^16,17^. The role of NR in the synthesis of NO during plant-pathogen interaction is well established^10^. NO is highly reactive and phytotoxic and is converted into a stable, mobile, less toxic form S-nitrosoglutathione (GSNO). NO's reaction with reduced glutathione (GSH) is catalyzed by S-nitroso glutathione reductase (GSNOR), class-III alcohol dehydrogenase, and controls intracellular GSNO level and NO homeostasis^11,18^. Glutathione-disulfide reductase (GSR) catalyzes the reduction of oxidized glutathione (GSSG) to GSH in the presence of reducing equivalent NADPH. Thus the activity of GSNOR balances the cellular RNS redox homeostasis^19^. GSNOR is also established as one of the key players in the plant immune system in Arabidopsis^18^.

As the information on NO homeostasis and the involvement of *NR* and *GSNOR* genes in wheat and leaf rust pathogen interaction is limited, we conducted (1) Genome-wide identification and *in-silico* analysis of *NR* and *GSNOR* family genes in wheat and (2) expression profiling of identified *TaNIA* and *TaGSNOR* family genes in response to leaf rust infection in wheat. Based on the genome-wide identification and expression profiling of *NR* and *GSNOR* genes and NO localization, nitrosothiol accumulation, NR, and GR activities, we found that NR and GSNOR family genes play a significant role in defence against the wheat leaf rust pathogen.

## Materials and methods

### Identification and physical mapping of wheat *NR*/*NIA* and *GSNOR* genes

To identify the putative candidate *NIA* and *GSNOR* genes from the wheat genome, the protein sequence of *NIA* and *GSNOR* candidate genes from *Arabidopsis,* rice and maize from TAIR (https://www.arabidopsis.org/index.jsp), the Rice Annotation Project database rap-db (https://rapdb.dna.affrc.go.jp) and Maize database (https://corncyc-b73-v4.maizegdb.org) respectively were used as BLAST queries. For NIA; amino acid sequences of *Arabidopsis* NIA1 (AT1G77760), *Arabidopsis* NIA2 (AT1G37130), rice *OsNIA1* (os02g0770800), and maize ZmNR (Zm00001d049995) and for GSNOR; amino acid sequences of *Arabidopsis* (AT5G43940) and rice (Os02g0815500) genes were used for BLASTP search against the fully annotated genome of wheat available at Ensemblplants (https://plants.ensembl.org/index.html). E-value was kept < 1E^−10^, and only wheat genes with E-value < 10^−10^ and identity > 50% were selected for further analysis. All the selected ID’s were used to download genomic (gDNA), cDNA, CDS, and protein (AA) sequences of each respective ID for further study. Conserved domains were searched in all the identified sequences using secondary databases, including InterPro (https://www.ebi.ac.uk/interpro)^20^ and PROSITE (https://prosite.expasy.org)^21^. All the identified wheat *NIA* and *GSNOR* genes were physically mapped on seven homeologous chromosomes groups by screening against information available in EnsemblPlants^22,23^ and IWGSC-URGI (https://wheat-urgi.versailles.inra.fr/) accordingly.

### Physiochemical properties and subcellular localization of *TaNIA* and *TaGSNOR*

The ProtParam tool of Expasy server (https://web.expasy.org/protparam/)^24^ was used to analyze different physiochemical properties of the wheat *TaNIA* and *TaGSNOR* genes. Physiochemical properties like molecular weight (MW), iso-electric point (pI), instability index (II), aliphatic index (AI), and grand average of hydropathicity (GRAVY) were predicted. The subcellular location of different *TaNIA* and *TaGSNOR* proteins was predicted by integrated web-server BUSCA (http://busca.biocomp.unibo.it/)^25^.

### Analysis of gene structure and regulatory motif variation and phylogenetic study

Gene Structure Display Server (GSDS v2.0) of Peking University, China, was used to analyze gene structure and exon/intron boundaries (http://gsds.cbi.pku.edu.cn/)^26^. Gene structure prediction in GSDS v2.0 uses genomic DNA and cDNA sequences as input. Amino acid sequences of all the identified wheat *TaNIA* and *TaGSNOR* genes were analyzed using MEME software version 5.0.5 ((http://meme-suite.org/meme_5.0.5/)^27^ to determine the variation in the regulatory motif present in the particular amino acid sequence of all the identified wheat TaNIA and TaGSNOR proteins. The MEME software run with parameters set at 15 AA, with a minimum width of 6 and a maximum of 50 amino acids.

Protein sequences of NIAs and GSNORs from wheat, Arabidopsis, maize and rice were aligned by the MUSCLE (multiple sequence alignment) options in MEGA X (v7) software. Sequence alignment was used to construct a phylogenetic tree based on the Maximum-Likelihood method with the Poisson substitution model, pairwise deletion, and uniform rates (https://www.megasoftware.net/)^28^.

### Scanning for cis-regulatory elements in the promoter region of genes

The 1500 bp region upstream to *TaNIA* and *TaGSNOR* gene sequences were analyzed in the plant CARE database (http://bioinformatics.psb.ugent.be/webtools/plantcare/html/) for identifying cis-regulatory elements^29^. The cis-regulatory elements associated with hormones, pathogen defence and stress responsiveness was identified.

### Potential miRNAs target and interaction network for genes

The genomic sequence *TaNIAs* and *TaGSNORs* were mined for the presence of simple sequence repeats (SSRs) using the tool SSRIT^30^. BatchPrimer3 v1.0^31^ software was used to design primer sequences for the identified SSR. The full-length gDNA sequences were also used as an input in psRNATarget server^32^ to identify the miRNAs targeting the particular *TaNIA* and *TaGSNOR* genes from a selection of updated wheat miRNAs libraries. The following parameters were set to identify the potential miRNAs: maximum expectation: 2.0, length for complementarity scoring (HSP size): 19, penalty G:U pairs: 0.5, seed region: 2–13 nt, and extra weight in seed region: 1.5. The desktop application of Cytoscape 3.5.1^33^ was used to construct the interaction network of miRNAs targeting *TaNIA* and *TaGSNOR* genes.

### In-silico expression analysis

The relative expression of all the identified *TaNIAs* and *TaGSNORs* were retrieved from public transcriptome data available in WheatEXP (http://www.wheat-expression.com/)] and wheat expression browser expVIP (http://www.wheat-expression.com).

### Analysis of Protein–protein interaction network and co-expression network:

The protein–protein interaction network (PIN), gene expressions network and prediction of interaction for TaNIAs and TaGSNORs were analyzed using the STRING tool (http://string-db.org/)^33^. Arabidopsis interactome data available at GeneMANIA server^34^ was also used for the analysis of protein–protein interaction.

### Homology modelling, structure evaluation, and structure alignment

Homology modelling method in the Swiss-Model server (https://swissmodel.expasy.org/)^35,36^ was used to predict 3D structures of wheat *TaNIA*s and *TaGSNOR*s. At the same time, the predicted models of wheat TaNIA and TaGSNOR proteins were rendered by UCSF CHIMERA 1.13.1 into different 3D coordinates^37^. Topology-independent comparison of all the identified TaNIA and TaGSNOR proteins was made by using CLICK server^38^. For Swiss-Model server validation, Ramachandran plots of modelled wheat TaNIA and TaGSNOR proteins were calculated by analyzing phi (Φ) and psi (Ψ) torsion angles and covalent bond quality using Swiss-Model server^35,36^.

### Plant material and pathogen inoculation

Seeds of two contrasting wheat lines, Chinese spring (CS), leaf rust susceptible line and Transfer (TR) introgression line in CS background with leaf rust resistance gene *Lr9* developed earlier^39^ and maintained by wheat breeding section of ICAR-IARI^40^ were used in the current study. The uredospores of leaf rust pathogen *Puccinia triticina* Eriks Pathotype 77–5 (121-R-63) were used to inoculate the two wheat genotypes.

### Inoculation and sampling

A set of 50 seeds of each variety were sown in 4-inch pots containing media having a 10:1 ratio of soil and FYM. Then pots were kept at optimum conditions for germination and ten days old seedlings were used for challenge inoculation. For inoculation, uredospore of leaf rust pathotype 77–5 (121-R-63) was thoroughly mixed with a surfactant and water to ensure the proper spread of inoculum onto the leaves. The inoculum was sprayed with a fine mist sprayer so that the fine droplets containing inoculum was distributed well onto the surface of leaves. After inoculation, seedlings were incubated in a humidity chamber for 48 hours^4^. After that, the seedlings were taken out from the humidity chamber and kept in a glasshouse under ambient conditions. The whole experiment was laid during rabi season in the glasshouse facility of the Division of Genetics, ICAR-IARI, New Delhi. Leaves were sampled at 0 HAI (Hours after Inoculation, un-inoculated), 24 HAI, 72 HAI, and 144 HAI from at least five different plants for further analysis.

### Visualization of NO using fluorescent microscope

Leaf samples from 0 HAI (Uninoculated) and 72 HAI were used for NO visualization. Leaves were cut into small pieces in 2 cm long segments using a surgical blade. The outer epidermal layer of the leaf segments from the mid leaf was removed using the surgical blade. The samples were then immersed in Diaminofluorescein-FM dye (5 µM DAF-FM in 20 mM HEPES–KOH with pH 7.5) in a watch glass for 30 min^41^. After 30 min, samples were taken out carefully and washed with HEPES–KOH buffer 2–3 times to remove the excess dye. The pieces were placed on a glass slide carefully with the help of a paintbrush and covered with a coverslip. Extra buffer was drained out with the use of Kim Wipes. The slides were visualized under a fluorescence microscope (Zeiss AXIOSKOP 2) at 495 nm excitation, and 515 nm emission wavelengths and the images were acquired^41^.

### Assay of NR and GR activity and estimation of S-nitrosothiol content

Fresh leaf samples weighing 1 g sampled from inoculated wheat seedlings of both lines at 0 HAI and 72 HAI was ground in 10 ml of extraction buffer (0.1 M phosphate buffer, pH 7.5, containing 0.5 mM EDTA and 1 mM ascorbic acid). The extract was passed through 4 layers of cheesecloth, and the filtrate was centrifuged for 20 min at 15,000 g, and the supernatant was used to carry out enzyme assays^42^. The assay mixture for the NR included 500 μl of 0.1-M KNO_3_, 1900 μl of phosphate buffer (0.1 M, pH 7.5), and 100 μl of 10-mM NADH. The reaction was initiated by the addition of 500 μl of enzyme extract into the assay mixture. The mixtures were incubated at 35 °C for 30 min. Further, 0.1 ml Zinc acetate was added to the incubated mixture, followed by the addition of 1.9 ml of 90% alcohol. The mixture was centrifuged at 3000 g for 10 min at room temperature. Subsequently, 1 ml of 1% (w/v) sulfanilamide solution and 1 ml of 0.02% (w/v) N-(1-naphthyl) ethylenediamine solution were added to the supernatant. Absorbance was recorded after 20 min at 540 nm^43^.

Activity of GR assay was assayed using the reaction mixture containing 66.67 mM potassium phosphate buffer (pH 7.5) and 0.33 mM EDTA (1 ml of 0.2 M buffer containing 1 mM EDTA), 0.5 mM DTNB in 0.01 M potassium phosphate buffer (pH 7.5) (0.5 ml of 3.0 mM), 66.67 µM NADPH (0.1 ml of 2.0 mM), 666.67 µM GSSG (0.1 ml of 20 mM), 0.1 ml enzyme extract and distilled water to make up a final volume of 3.0 ml. Reaction was started by adding 0.1 ml of 20.0 mM GSSG (oxidized glutathione). The increase in absorbance at 412 nm was recorded for 60 s. The activity was expressed as total absorbance (∆A_412_) per mg protein per min^44^.

The content of S-nitrosothiol was estimated in leaf samples at 0 HAI and 72HAI. Fresh leaf sample (0.5 g) was homogenized using liquid nitrogen, followed by the addition of 1.5 ml of extraction buffer. The extract was centrifuged at 13,800 X g/12,500 rpm for 25 min at 4 °Celsius. Reaction mixture consisting of 250 µl of supernatant and 50 µl ammonium sulfonate was incubated for 2 min, followed by the addition of 300 µl sulfanilamide, 300 µl HgCl_2,_ and 300 µl NEDD. All the steps were performed in dark conditions. The OD value was recorded at 540 nm^45^.

### Isolation of total RNA, cDNA Preparation

The leaf samples collected at different time intervals (0 h after inoculation (HAI), 24 HAI, 72 HAI, and 144 HAI) and frozen in liquid nitrogen was used for RNA extraction. Total RNA was extracted using RNAeasy plant mini kit (Qiagen Inc., Chatsworth CA 91,311, USA, Cat No: 749040), followed by on-column DNA digestion with DNase I (Qiagen Science, Maryland, USA) to remove DNA contamination. RNA was quantified using a thermo nanodrop 2000c spectrophotometer, and purity was confirmed by checking the ratio of A260/A280. RNA was reverse transcribed using a superscript III reverse transcriptase kit (Invitrogen, Life Technologies, USA).

### Primer Designing and qRT-PCR analysis

cDNA sequences of the identified *NIA* and *GSNOR* genes were used for manual primer designing. The specificity of primer sequences was confirmed by the Primer blast tool of NCBI. Gene-specific primers (Supplementary Table 1), cDNA and SYBR® Green Master Mix (Applied Biosystems, USA) were used for qPCR analysis in a real-time detection system (CFX96 Touch real-time PCR detection, BIO-RAD life sciences). *TaActin* (housekeeping gene) was used as an internal control for normalization of the data for each transcript^46^, and level of expression or fold change in expression of *TaNIA* and *GSNOR* genes were analyzed using the 2^−ddCt^ method^47^.

### Compliance with ethical standards

The experimental research on plants complied with relevant institutional, national, and international guidelines and legislation.

## Results and discussion

### Identification and physical mapping of *TaNIA* and *TaGSNOR* genes

By genome-wide analysis, 9 NIA and 3 GSNOR orthologous genes were identified in the wheat genome. The identified genes were named *TaNIA1*-4a to *TaNIA9-7d* and *TaGSNOR1-6A to TaGSNOR3-6D,* respectively, based on their position on a particular chromosome. The Ensemble gene ID, gene sequence length in base pair (bp), length of amino acid, chromosome location, coordinates, number of splice variants, and sub-cellular location of all the identified *TaNIA* and *TaGSNOR* genes are listed in Table 1. The length of coding sequence (CDS) in *TaNIA* genes ranged from 2691 (*TaNIA1-4a*) to 3366 bp (*TaNIA4-6b*), and the corresponding protein’s amino acids ranged from 866 aa (*TaNIA8-7a*) to 914 aa (*TaNIA2-6a*, *TaNIA4-6b*, *TaNIA6-6d*) (Table 1). In *TaGSNOR* genes length of the coding sequence (CDS) varied from 1367 bp (*TaGSNOR1)* to 1629 bp (*TaGSNOR3)*. The protein length for all the *TaGSNORs* was 381 aa (Table 1).

**Table 1:** Details of *TaNIA* and *TaGSNOR* genes with their gene ID, length, chromosome location, coordinates, and splice variants.

Gene name	Ensemble ID	Splice variant	Splice selected	Strand	Coordinates	Exon	Coding exon	bp	aa	Genome location	Domain
***TaNR/NIA***											
*TaNIA1-4a*	TraesCS4A02G376700	1	TraesCS4A02G376700.1	F	651,365,942–651,370,213	3	3	2691	896	4A: 651,365,942	PLN02252
*TaNIA2-6a*	TraesCS6A02G017500	2	TraesCS5A02G138900.1	F	8,694,483–8,700,180	3	3	3246	914	6A: 8,694,483	PLN02252
*TaNIA3-6a*	TraesCS6A02G326200	1	TraesCS5A02G185800.1	R	559,523,404–559,526,788	7	7	3089	899	6A: 559,523,404	PLN02252
*TaNIA4-6b*	TraesCS6B02G024900	1	TraesCS5A02G391900.1	F	15,128,191–15,134,280	3	3	3366	914	6B: 15,128,191	PLN02252
*TaNIA5-6b*	TraesCS6B02G356800	1	TraesCS5B02G138200.1	R	625,241,370–625,244,779	3	3	3061	897	6B: 625,241,370	PLN02252
*TaNIA6-6d*	TraesCS6D02G020700	1	TraesCS5B02G183900.1	R	8,149,929–8,155,961	6	6	3321	914	6D: 8,149,929	PLN02252
*TaNIA7-6d*	TraesCS6D02G306000	1	TraesCS5B02G396800.1	F	414,622,355–414,625,758	3	3	3048	895	6D: 414,622,355	PLN02252
*TaNIA8-7*a	TraesCS7A02G078500	1	TraesCS5D02G152500.1	F	43,180,494–43,185,382	3	3	2749	866	7A: 43,180,494	PLN02252
*TaNIA9-7d*	TraesCS7D02G073700	1	TraesCS5D02G190900.1	R	43,212,472–43,217,253	6	6	2849	871	7D: 43,212,472	PLN02252
***TaGSNOR***											
*TaGSNOR-1*	TraesCS6A02G386600	1	TraesCS6A02G386600.1	F	603,279,456–603,282,956	1367	381	9	9	6A: 603,279,456	Alcohol_DH_class_III
*TaGSNOR-2*	TraesCS6B02G425700	1	TraesCS6B02G425700.1	F	694,401,891–694,405,637	1529	381	9	9	6B: 694,401,891	Alcohol_DH_class_III
*TaGSNOR-3*	TraesCS6D02G371200	1	TraesCS6D02G371200.1	F	456,554,723–456,558,968	1629	381	9	9	6D: 456,554,723	Alcohol_DH_class_III

PLN02252; nitrate reductase [NADPH], and Alcohol_DH_class_III; S-(hydroxymethyl) glutathione dehydrogenase.

**Figure 1:**
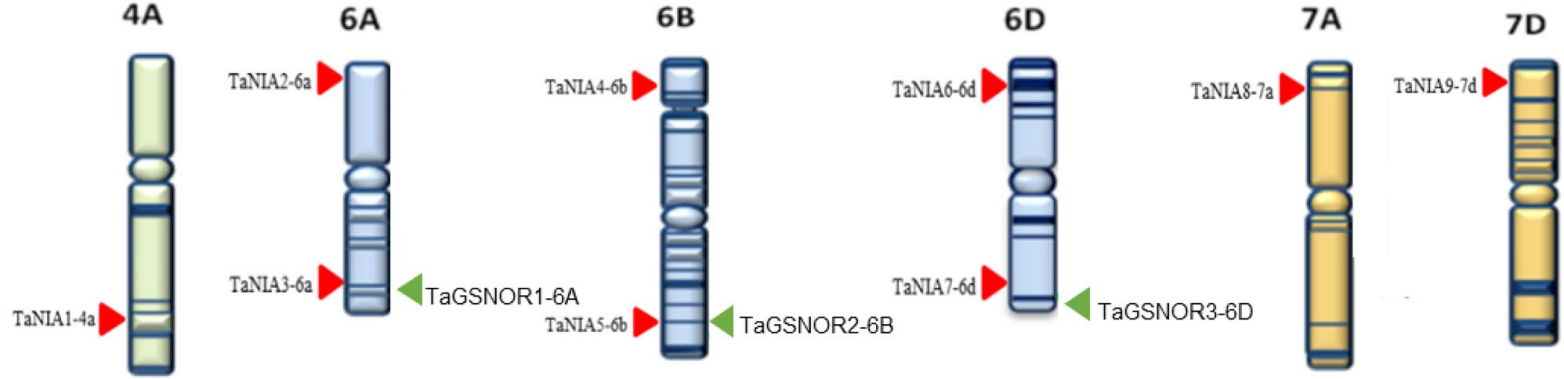
Distribution of the identified 9 *TaNIA* genes (red arrows) and 3 *TaGSNOR* genes (green arrows) in the A, B and D homoeologous genomes of wheat. Image created in WGSC website (https://wheat-urgi.versailles.inra.fr/).

The nine *TaNIA* genes were mapped on three different wheat homeologous groups; 4, 6 and 7. However, all three *TaGSNOR* genes were located on homeologous group 6. Out of the nine *TaNIA* genes, one gene was mapped on chromosome 4A, 6 *TaNIA* genes were mapped on chromosome 6A, 6B, and 6D with two genes on each chromosome, and the remaining two genes were mapped on chromosome 7A and 7D of group 7 (Fig. 1). Three homeologous chromosomes of group 6; 6A, 6B, and 6D each carried one copy of three identified *TaGSNOR* genes. Homoeologs showed a high level of similarity (> 98%) among each other in both *TaNIAs* and *TaGSNORs*. Some of the genes did not have three homeologs; for example, *TaNIA1-4a* did not have homoeologs on chromosomes 4B and 4D.

### Physiochemical properties of TaNIA and TaGSNOR proteins

The predicted physicochemical properties of TaNIA and TaGSNOR proteins are listed in Table 2. The calculated molecular weight (Mw) of TaNIA proteins ranged from 96,954.41 g/mol (TaNIA8-7a) to 1,01,845.65 g/mol (TaNIA2-6a) and the isoelectric point (pl) ranged from 6.3235 (TaNIA2-6a) to 7.1944 (TaNIA5-6b). Out of the nine TaNIA proteins, only 3 have a stable nature, while the remaining six proteins showed an Instability index higher than 40. Similarly, the molecular weight (MW) of TaGSNOR proteins varied from 40,611.65 g/mol (TaGSNOR1) to 40,700.70 g/mol (TaGSNOR2), and the iso-electric point of TaGSNOR proteins are lies around 6.7 to 6.9. All the TaGSNOR proteins are stable, having an instability index of less than 40. The aliphatic index of all TaNIA and TaGSNOR proteins was in the range of 77.81-78.7 and around 85, respectively, suggesting their higher thermostability at a wide range of temperatures. The predicted GRAVY score of all TaNIA proteins is around -0.3, while for TaGSNOR proteins ranged from 0.035 (TaGSNOR2) to 0.049 (TaGSNOR1), suggesting that all are hydrophilic and more likely globular in structure (Table 2). The predicted subcellular localization of TaNIAs were in the cytosol, chloroplast, and nucleus (Table 3), and TaGSNORs in the cytosol.

**Table 2:** Details of *TaNIA* and *TaGSNOR* genes proteins including average residue weight g/mol, charge, isoelectric point, molecular weight, theoretical PI, instability index, aliphatic index, grand average of hydropathicity (GRAVY) and stability.

Protein	Mol weight g/mol	charge	Isoelectric point	Theoretical pl	Instability index	Aliphatic index	GRAVY	Stable
***TaNR/NIA***								
TaNIA1-4a	99,942.08	8	6.905	6.45	38.97	78.04	-0.349	Yes
TaNIA2-6a	1,01,845.65	-3	6.3235	5.95	43.95	78.53	-0.384	No
TaNIA3-6a	99,127.86	4	6.7538	6.32	46.51	78.16	-0.302	No
TaNIA4-6b	1,01,661.51	-1	6.4456	6.05	43.32	78.22	-0.379	No
TaNIA5-6b	99,123.03	9.5	7.1944	6.81	47.74	78.34	-0.315	No
TaNIA6-6d	1,01,767.51	-2	6.3848	6	41.99	78.11	-0.394	No
TaNIA7-6d	98,926.63	3.5	6.7299	6.3	48.45	78.29	-0.306	No
TaNIA8-7a	96,954.41	7.5	6.9509	6.51	37.91	77.81	-0.391	Yes
TaNIA9-7d	97,244.85	3	6.667	6.23	38.4	78.7	-0.359	Yes
***TaGSNOR***								
TaGSNOR-1	40,611.65	3.0	6.9537	6.55	23.79	85.64	0.049	Yes
TaGSNOR-2	40,700.70	2.0	6.7897	6.37	24.4	85.38	0.035	Yes
TaGSNOR-3	40,656.69	2.0	6.7897	6.37	23.25	85.64	0.044	Yes

**Table 3:** Subcellular location of all the identified *TaNIA* and *TaGSNOR* genes.

Protein accession	GO-id	GO term	Score	Features
***TaNIA***				
TaNIA1-4a	GO:0,005,737	C:cytoplasm	0.7	Nitrate reductase
TaNIA2-6a	GO:0,009,507	C:chloroplast	1	Nitrate reductase
TaNIA3-6a	GO:0,005,634	C:nucleus	1	Nitrate reductase
TaNIA4-6b	GO:0,009,507	C:chloroplast	1	Nitrate reductase
TaNIA5-6b	GO:0,005,634	C:nucleus	1	Nitrate reductase
TaNIA6-6d	GO:0,009,507	C:chloroplast	1	Nitrate reductase
TaNIA7-6d	GO:0,005,634	C:nucleus	1	Nitrate reductase
TaNIA8-7a	GO:0,005,737	C:cytoplasm	0.7	Nitrate reductase
TaNIA9-7d	GO:0,005,737	C:cytoplasm	0.7	Nitrate reductase
***TaGSNOR***				
TaGSNOR-1	GO:0,005,737	C:cytoplasm	0.7	–
TaGSNOR-2	GO:0,005,737	C:cytoplasm	0.7	–
TaGSNOR-3	GO:0,005,737	C:cytoplasm	0.7	–

### Phylogenetic relationship, gene structure and regulatory motifs

**Figure 2:**
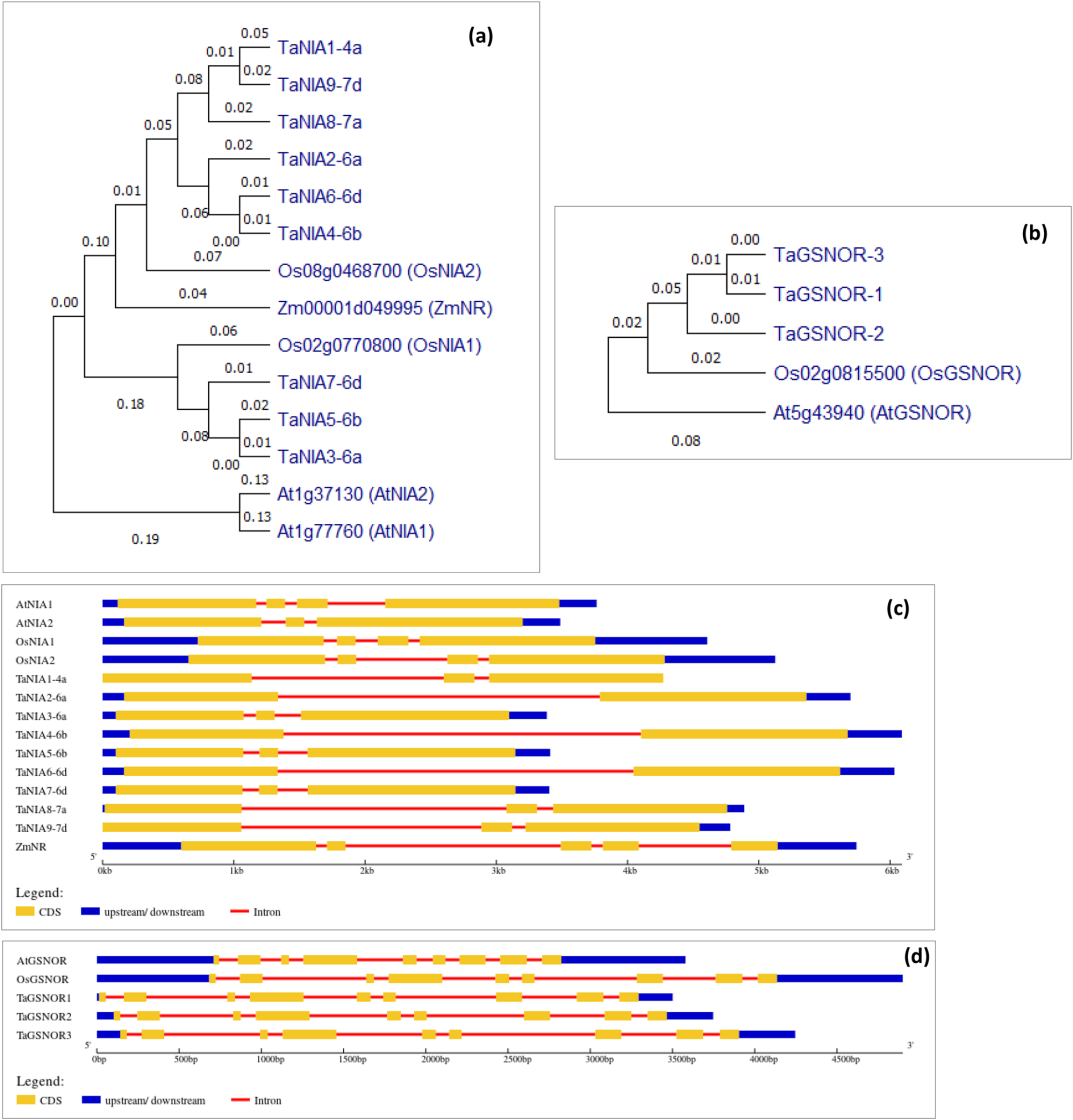
Phylogenetic relationship and gene structure of wheat *TaNIA* and *TaGSNOR* genes (**a**, **b**) Phylogenetic relationship of putative TaNIA TaGSNOR proteins and homologues from Arabidopsis, rice, and maize. (**c**, **d**) Illustration of gene structure of wheat *TaNIA* and *TaGSNOR* genes, showing the distribution of exons/introns, and exon phase. (**a**, **b**) Were created in in MEGA X (v7) software (https://www.megasoftware.net/). (**c**, **d**) Were created in Gene Structure Display Server (GSDS v2.0) (http://gsds.cbi.pku.edu.cn/).

*TaNIA1-4a*, *TaNIA2-6a*, *TaNIA4-6b*, and *TaNIA6-6d*, *TaNIA8-7a,* and *TaNIA9-7d* comprise the first group, along with two Arabidopsis genes (*AtNIA1* and *AtNIA2*) and one maize gene (*ZmNR*), while *TaNIA3-6a*, *TaNIA5-6b*, and *TaNIA7-6d* form the second group, along with rice NIA gene *OsNIA1* (Fig. 2a). *TaGSNOR* genes were clustered with *GSNOR* genes from Arabidopsis and rice (*AtGSNOR* and *OsGSNOR*) (Fig. 2b).

Comparative analysis of CDS and the genomic DNA sequences revealed the gene structure (exon/intron numbers, boundaries, length etc.) of the identified *TaNIA and TaGSNOR* genes (Fig. 2c). We found that *TaNIA2*, *TaNIA4,* and *TaNIA6* have only one intron, and other genes have two introns. The exon\intron structure, intron phase, intron number, and exon length of *TaNIA* genes share a similar pattern (Fig. 2d). Similarly, all three *TaGSNOR* genes had an equal number of introns, the intron phase, exon length and shared identical patterns (Fig. 2d).

The conserved motifs analyses predicted 15 distinct conserved regulatory motifs in *TaNIA* proteins and eight distinct conserved motifs in *TaGSNOR* proteins (Table 4, Supplementary Fig. 1, 2). The motif prediction depicted that the location of the conserved motif is highly conserved in both the gene families. All the *TaNIA* proteins have an NR conserved domain, and the *TaGNORs* proteins have the S-(hydroxymethyl) glutathione dehydrogenase domain (Table 1).

**Table 4:** Details of discovered motif in identified *TaNIA* and *TaGSNOR* genes.

Sl. NO	Discovered motif	Log Likelihood Ratio	Information Content	Relative Entropy	Bayes Threshold
***TaNA/TaNIA***					
1	WIVVHGHVYDCTAFLKDHPGGADSILINAGSDCTEEF	2487	118.3	112.1	9.08414
2	IWNLMGMMNNCWFKVKINVCRPHKGEIGLVFEHPTQPGNQTGGWMARQKH	1368	210	219.2	10.9685
3	AWWYKPEYIINELNTNSVITTPGHDEILPINAFTTQRAYTMKGYAYAGGG	1293	206.8	207.2	10.2544
4	DIGHSDSAREMMEKYHIGEIDASTIPAKR	1411	83.8	78.3	8.43576
5	CTLDIPEKPNKYGRYWCWCFWSVDVEVLDLLGAKEVAVRAWDQAQNTQPE	1274	204	204.2	10.2544
6	MICGGSGITPMYQVIQAVLRDQPEDETEMHLVYANRSEDDILLRDELDRW	1253	205.1	200.9	10.2544
7	IRLTGKHPFNCEPPLARLMHHGFITPAPLHYVRNHGPVPRGDWSTWTVEV	1260	206	201.9	10.2544
8	QNGEPLLPDHGFPVRVIIPGCIGGRMVKWLTRIVVTAAESDNYYHFKDNR	1240	201.8	198.7	10.9685
9	PVTLVCAGNRRKEQNMVRQTAGFNWGAAGVSTSVWRGARLRDVLRRCGIM	1235	203.1	198	10.2544
10	KKELSHDVRLFRFALPSSDQVLGLPVGKHIFVCATIDGKLCMRAYTPTSM	1216	200.9	195	10.2544
11	AAEYPERLKVWYVIDQVKRPEDGWRFSVGFVTEDILRAHVPEGGD	1004	170.6	160.9	8.53403
12	HTTDDKQFTMSEVRKHGSKDS	1026	53.2	51	9.19922
13	VDEIGQFELLVKVYFKDEHPKFPSGGLMTQYLESLQLGSC	923	153.6	148	10.2997
14	IKILQFLVPLAILGLAVAIRMYTKSE	766	83.3	78.9	9.43211
15	CGPPPMIKFAISPNLEKMKYDMANSFISF	690	109.8	110.7	11.6188
***TaGSNOR***					
1	QVPWLVEKYMNKEIKVDEYITHNMNLTDINKAFDLLHEGGCLRCVLAMEH	455	216.1	219	8.81931
2	MMSDRKSRFSVNGKPIYHFMGTSTFSQYTVVHDVSVAKINPQAPLDKVCL	447	216.1	214.9	8.8193
3	YEANKPLVVEDVQVAPPQAGEVRIKILSTALCHTDYYTWSGKDPEGLFPC	441	216.1	212.1	8.81931
4	TEFVNPKDHDKPIQQVLVDLTDGGVDYSFECIGNVSIMRAALECCHKGW	432	210.9	207.6	8.82366
5	HEAAGIVESVGEGVTDVQPGDHVIPCYQAECKDCKMCNSGKTNLCGKVR	417	209.9	200.7	8.82366
6	WNTAKVEAGSIVAVFGLGTVGLAVAEGAKSAGASRIIGIDIDTKKFDVAK	399	214.3	191.8	8.81931
7	SGQEIATRPFQLVTGRVWKGTAFGGFKSR	252	125.3	121.3	8.90798
8	MASSTQGQVITCKAA	128	64.8	61.3	8.96421

### Cis-regulatory elements in the promoter region

*TaNIA* and *TaGSNOR* cis-regulatory elements investigation revealed potential cis-acting regulatory elements (CAREs) such as responsive to various hormones and defence and stress-responsive elements. Hormone related elements include MeJA-responsiveness (MeJARE), abscisic acid responsiveness (ABRE), and salicylic acid responsiveness (SARE). Some other detected features were related to development responsiveness (GARE, ARE, Meristem expression) and abiotic stress response (DRE, MYB, LRE) (Supplementary Table 2).

### Gene-specific SSRs and network of miRNAs targeting *TaNIA and TaGSNOR genes*

In *TaNIA* genes, 9 SSR motifs were identified with tri-nucleotide repeat (NNN)n motifs present in high frequency (in 8 out of 9 genes) and di-nucleotide (NN)n repeat motifs (only one gene) (Supplementary Table 3). No SSR motifs were found for *TaGSNOR* genes. In addition to the discovery of SSR motifs, a set of 27 wheat miRNAs (Tae-miRs) targeting nine different *TaNIA* genes and a set of 5 wheat miRNAs (Tae-miRs) targeting three different *TaGSNOR* genes were also predicted by the psRNATarget server. The identified Tae-miRs belongs to 13 and 3 different miR families (Supplementary Table 4, Supplementary Fig. 3).

### Protein–protein interaction network and co-expression analysis

**Figure 3:**
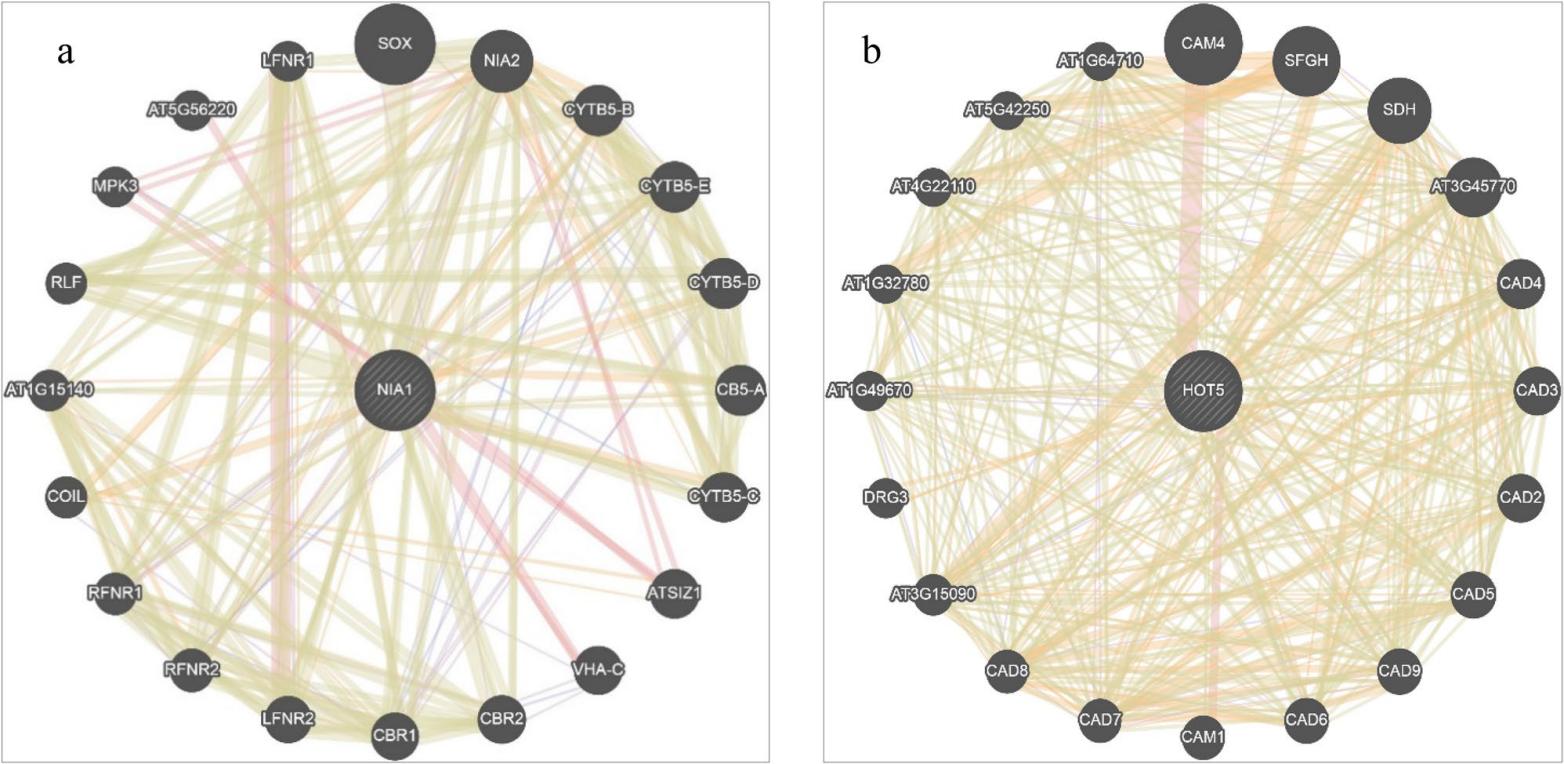
Predicted protein protein interaction network (PPIN) of TaNIA proteins (**a**) and TaGSNOR proteins (**b**) with other proteins. Images were created in GENEMANIA server (http://www.genemania.org).

The NR protein, Traes_6DS_69570DBE2.1, showed a maximum bit score among all TaNIA proteins was selected for predicting the PPIN. The expected network revealed that the Traes_6DS_69570DBE2.1 protein, similar to the TaNIA protein, interacts with many uncharacterized proteins; two were electron carrier proteins (Traes_3B_EB4EF9F7C2.1 and Traes_3B_EB4EF9F7C2.1) (Fig. 3a). The S-(hydroxymethyl) glutathione dehydrogenase protein, Traes_6DL_FD8A6A45F.1, showed the bit score 775.8 was selected for prediction of PIN. The predicted network revealed that the Traes_6DL_FD8A6A45F.1 interact with many different types of proteins, viz., aldehyde dehydrogenase family proteins, S-formyl glutathione hydrolase, and other uncharacterized proteins depicted in (Fig. 3b).

The gene expression analysis by the STRING tool also revealed similar results as shown by PPIN (Supplementary Fig. 4). Predicted association of TaNIA, Traes_6DS_69570DBE2.1 based on observed co-expression of homologs in other species indicated the co-regulation of CB5LP—Cytochrome B5-like protein; NADH-cytochrome b5 reductase-like protein; Ferredoxin–nitrite reductase etc. (Supplementary Fig. 4a). Predicted association of *TaGSNOR*, Traes_6DL_FD8A6A45F.1 based on observed co-expression of homologs in other species indicated the co-regulation of S-formyl glutathione hydrolase, Serine hydrolase, Alcohol dehydrogenase class-3 etc. (Supplementary Fig. 4b).

### Homology modelling, structure evaluation, and structure alignment of TaNIA and TaGSNOR proteins

**Figure 4:**
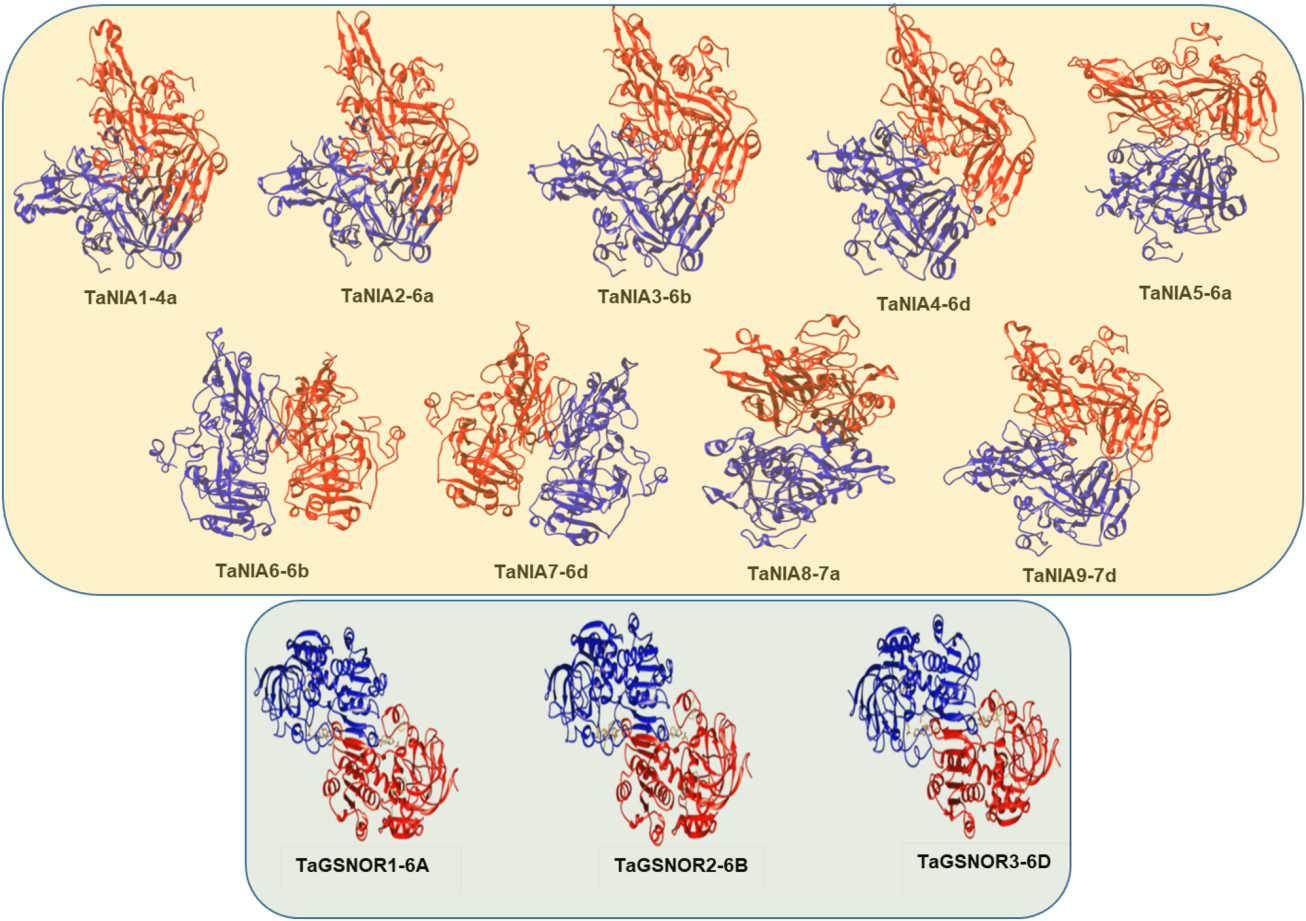
Homology modeling of *TaNIA* (**a**) and *TaGSNOR* (**b**) proteins constructed using automated Swiss-Model server by comparing template 2bih.1. A (Nitrate reductase [NADPH domain]) and 4dl9.1. A (Alcohol dehydrogenase class III) respectively and visualized in UCSF CHIMERA to generate 3D images (https://www.cgl.ucsf.edu/chimera/).

**Figure 5:**
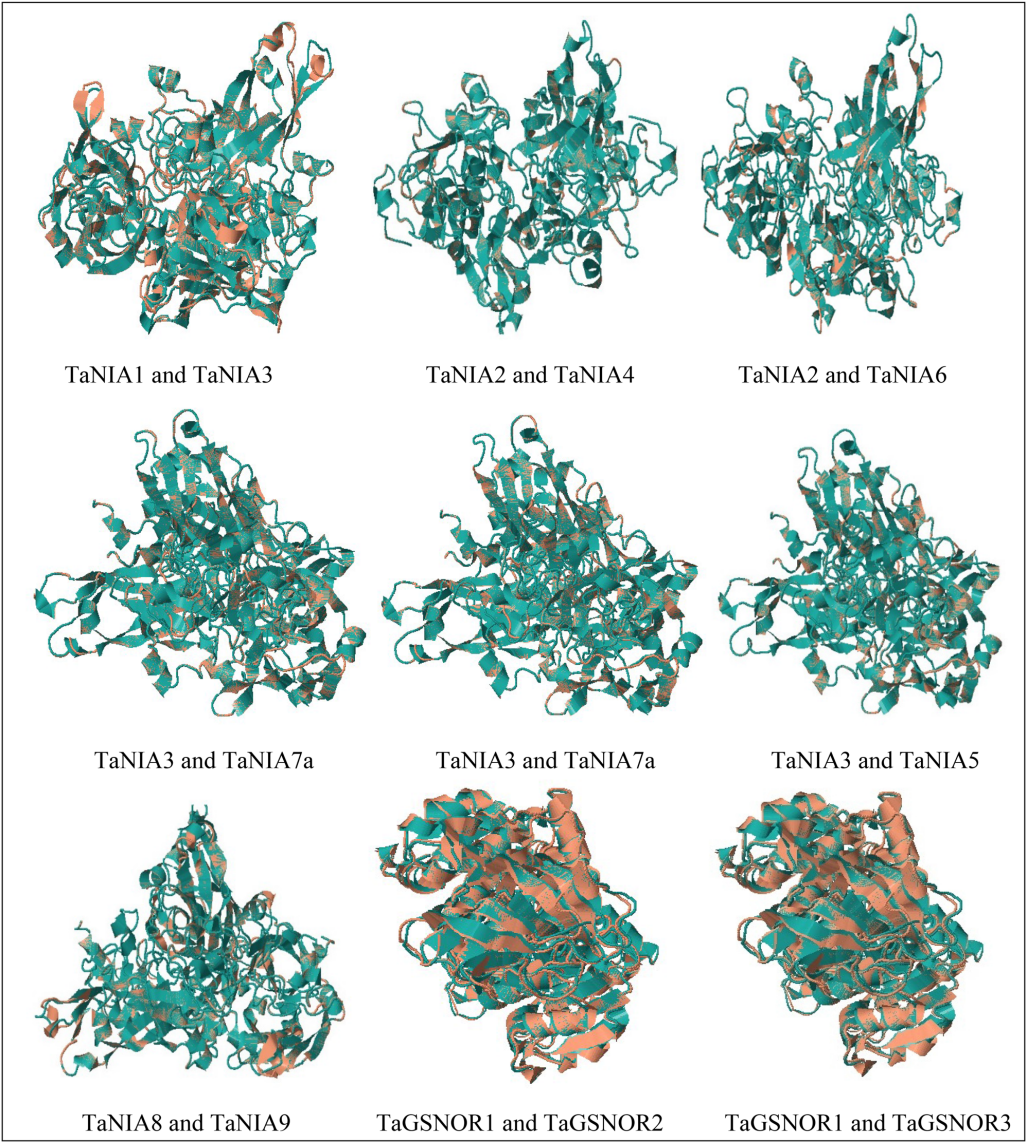
Structural overlapping of TaNIA and TaGSNOR proteins predicted using CLICK server (http://cospi.iiserpune.ac.in/click/). The alignment of pair of proteins in CLICK identify structural similarity by superimposing the 3D structures, independent of their topology. Images were created in CLICK server (http://cospi.iiserpune.ac.in/click/).

The Protein 3D structure of nine TaNIA proteins was modelled using the homology modelling-based method. Homolog templates were selected from the Protein Database (PDB) based on the sequence alignment among target and template proteins identified on the swiss model server (Supplementary Table 5, Fig. 4). The TaNIA proteins were compared with the templates 2bih.1.A and 1cnf.1.A of PDB, which are the structure of nitrate reductase NADPH active site and cytochrome b reductase fragment of nitrate reductase respectively^48^. The per cent sequence identity of the target TaNIA proteins with template protein, template protein ID, QMEAN, template description, oligo state, and Ramachandran favoured per cent are shown in Supplementary Table 5. The predicted 3D structure of TaNIA proteins construed by an automated Swiss-Model server was further visualized in UCSF CHIMERA. The same procedure was followed to identify the template protein on the swiss model server for TaGSNOR proteins. The TaGSNOR proteins were compared with the template 4dl9.1. A of PDB, which is the structure of Alcohol dehydrogenase class III. The per cent sequence identity of the target TaGSNOR proteins with template protein, template protein ID, QMEAN, template description, oligo state, and Ramachandran favoured per cent is shown in Supplementary Table 5 and Supplementary Fig. 5. Predicted 3D structures of TaGSNOR proteins construed using an automated Swiss-Model server were further visualized in UCSF CHIMERA. Alignment of different TaNIA/TaGSNOR protein pairs includes other details like structure overlap (%), RMSD, fragment score, topology score, match the size, identical residues, and heuristics, and Z-score values are given in Supplementary Table 5. The output of structural alignment of different proteins is shown in Fig. 5. Structural overlapping of TaNIA and TaGSNOR proteins were predicted using CLICK server (http://cospi.iiserpune.ac.in/click/). The alignment of pair of proteins in CLICK identify structural similarity by superimposing the 3D structures, independent of their topology. Details of overlapping of TaNIA and TaGSNOR proteins are given in Supplementary Table 6, and Fig. 5. The structural overlap was 100% among TaNIA3:TaNIA7, and TaNIA3:TaNIA5. Among NIAs, the similarity was least (98.47%) in TaNIA1 and TaNIA3. The TaGSNORs were structurally similar (99%) to each other.

### Expression analysis of *TaNIA* and *TaGSNOR* genes in wheat seedlings and insilico expression profiling

Expression of *NIA*s and *GSNOR*s were retrieved from exVIP (Supplementary Fig. 6) from experiments with wheat inoculated with stripe rust pathogen CYR31 and powdery mildew pathogen. The biotic stresses up-regulated Ta NIAs' expression; however, the magnitude of expression was highest in *NIA2*, *NIA4,* and *NIA6*. In the case of GSNORs, there was a general up-regulation in response to biotic stress. *GSNOR2* and *GSNOR3* were highly up-regulated by stripe rust inoculation.

### Expression profiling of *TaNIAs* and *TaGSNORs* in response to leaf rust inoculation

**Figure 6:**
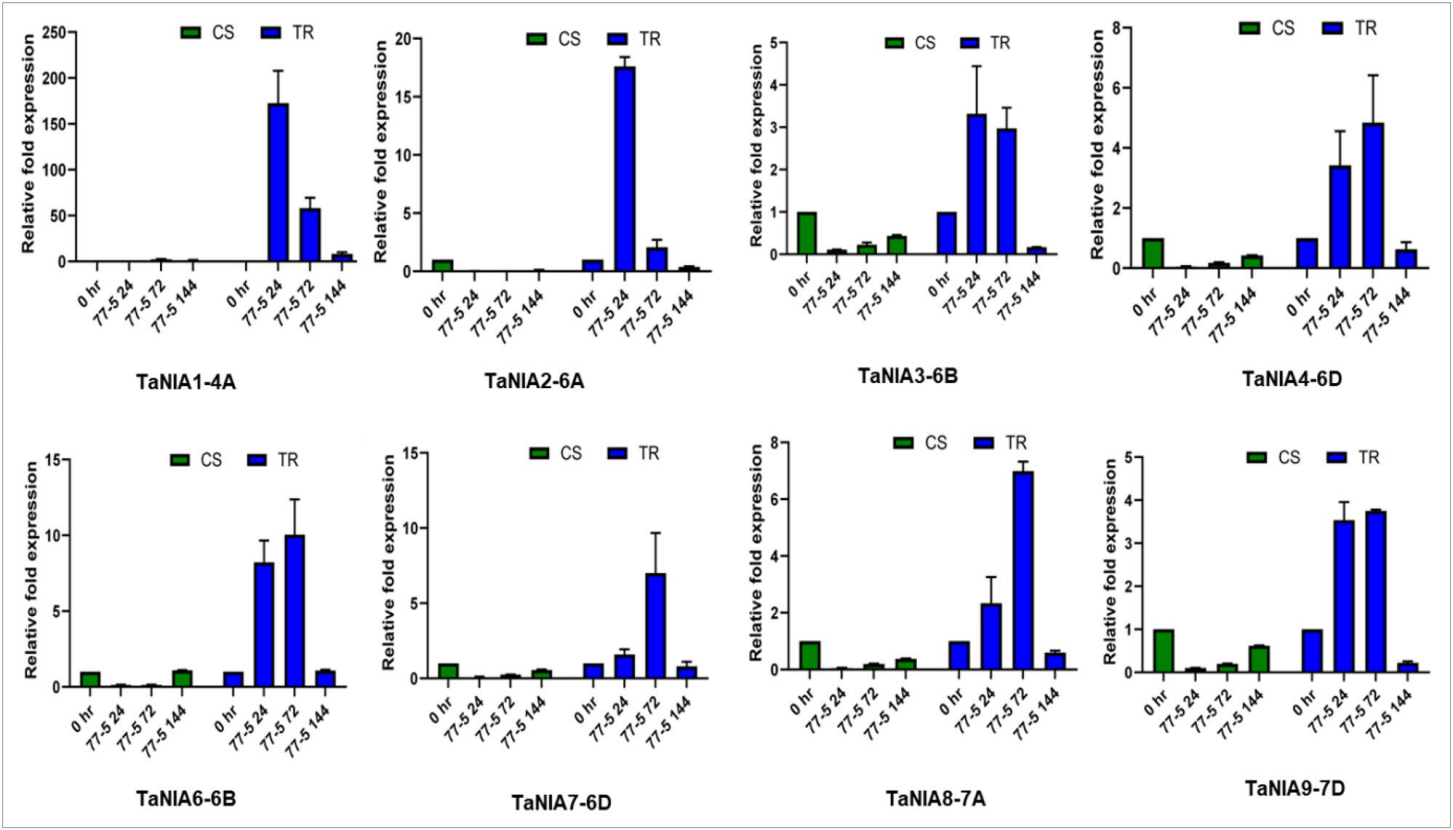
Expression analysis of *TaNIA* genes in leaves of wheat genotypes Chinese spring (CS) (susceptible) and transfer (TR) (resistant line) inoculated with leaf rust pathotype 77-5. Samples were collected at different time intervals viz., 0 h after inoculation (HAI), 24HAI, 72HAI and 144HAI. Values are means (± SE) of 3 biological replicates. Graphs were created in Graph Pad prism 8 (www.graphpad.com).

**Figure 7:**
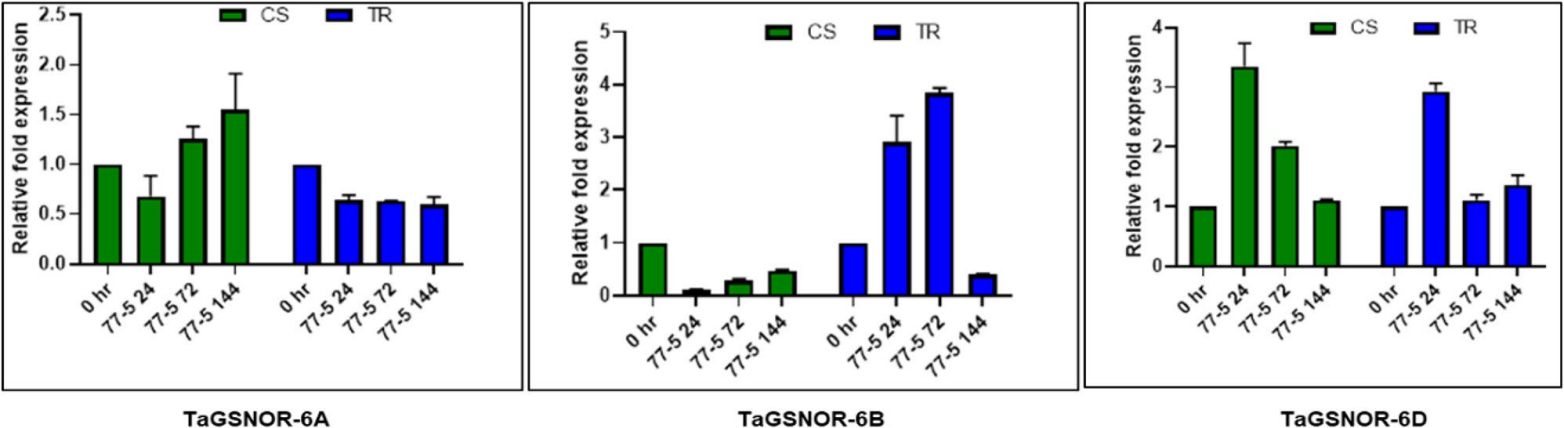
Expression analysis of *TaGSNOR* genes in leaves of wheat genotypes Chinese spring (CS) (susceptible) and transfer (TR) (resistant line) inoculated with leaf rust pathotype 77-5. Samples were collected at different time intervals viz., 0 h after inoculation (HAI), 24HAI, 72HAI and 144HAI. Values are means (± SE) of 3 biological replicates. Graphs were created in Graph Pad prism 8 (www.graphpad.com).

The expression response of *TaNIA* and *TaGSNOR* genes were validated by quantitative RT-PCR analysis. Total RNA extracted from CS and TR infected with leaf rust pathotype 77–5 (121-R-63) at different time points, 0 HAI, 24HAI, 72HAI, and 144HAI was used for the analysis. Out of the 9, NIAs analyzed, only 8 of them have amplification except for *TaNIA5*. In response to leaf rust infection, the expression of all of the NIAs was up-regulated. Expression of *TaNIA1*, *TaNIA2,* and *TaNIA3* was up-regulated to the maximum level at 24 h after inoculation. When we compared the relative fold expression, the *TaNIA1* gene showed maximum up-regulation (approx. 250fold) at 24HAI, followed by a steady decrease in expression at other time points. Expression of *TaNIA4*, *TaNIA6,* and *TaNIA7* was up-regulated at both 24HAI and 72HAI; however, maximum up-regulation occurred after 72HAI. *TaNIA7* and *TaNIA8* showed another distinct pattern in gene expression. In both, these genes expression was conspicuously up-regulated at 72HAI only (Fig. 6). Expression of *TaGSNOR1* showed no or negligible changes in expression w.r.t treatments. Whereas *TaGSNOR2* was highly up-regulated by leaf rust pathogen, 24 and 72 h after inoculation, followed by a sharp decrease in expression at 144 h of inoculation (Fig. 7).

### Visualization of NO using fluorescent microscope

**Figure 8:**
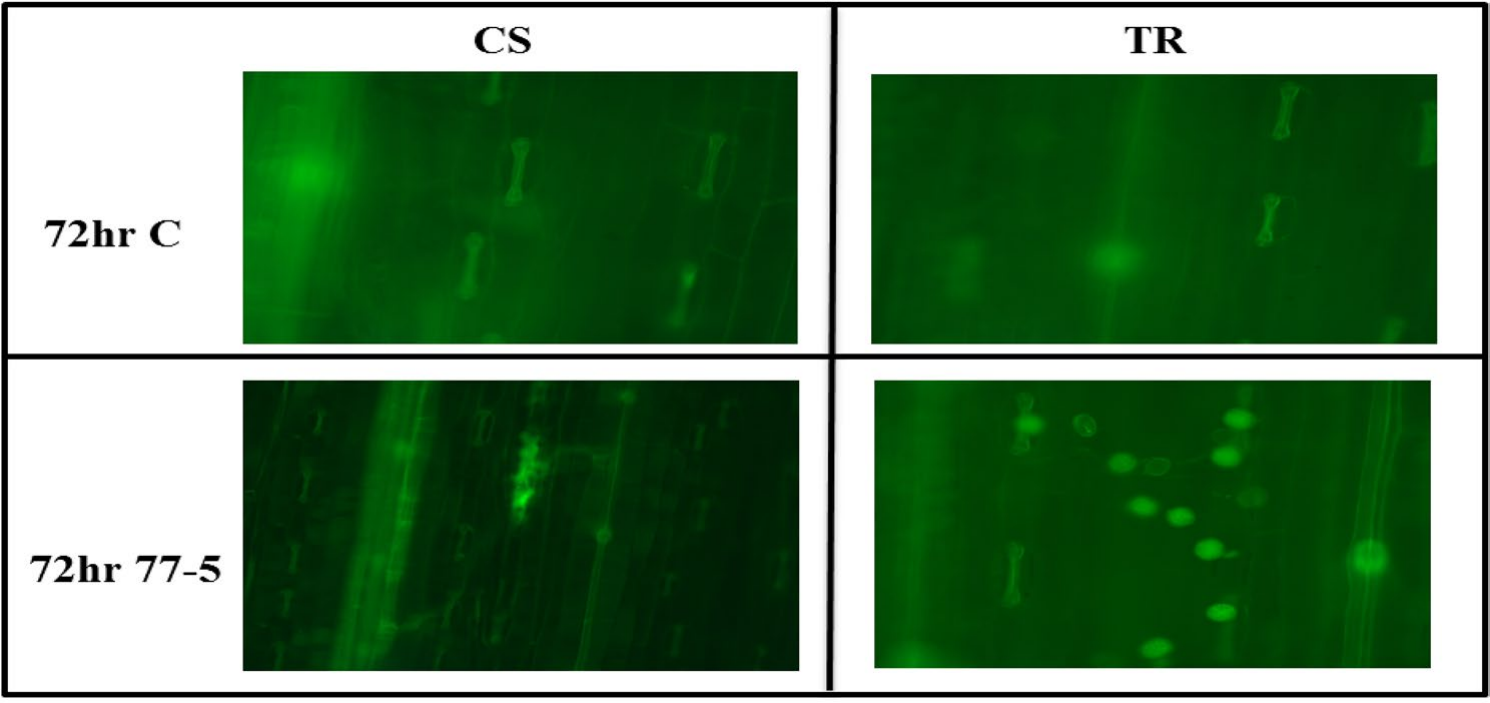
Effects of leaf rust pathogen on NO production; NO production is shown as green fluorescence in representative leaves. NO production was by fluorescence microscopy in the leaves with DAF-FM DA. Wheat genotypes Chinese spring (CS) (susceptible) and transfer (TR) (resistant line) inoculated with leaf rust pathotype 77-5. Samples were collected at 72 h after inoculation (HAI) and from uninoculated plants (C).

Based on the previous reports^49^ 72HAI was selected to study NO localization. After inoculation with pathotype 77–5, the leaves of the two combinations were sampled at 72 h after inoculation for NO labelling, and the results are shown in Fig. 8. Green fluorescence could be observed in stomatal guard cells for compatible and incompatible combinations, indicating that trace amounts of NO were produced. Over time, the NO fluorescence in the mesophyll cell around the infected site gradually intensified, and the fluorescent area expanded in compatible interaction. However, no lesions were formed in incompatible interaction, and NO fluorescence was limited to spherical structures suspected to be fungal spores. These results showed that NO production in the interaction between wheat plants and *P. triticina* varied widely among different combinations, with the incompatible combination producing NO in the early stage after inoculation and limited NO production observed at later time points, suggesting that NO may be associated with HR defence in wheat plants induced by *P. triticina*.

### Enzyme assay of NR and GR and Estimation of S-nitrosothiols

**Figure 9:**
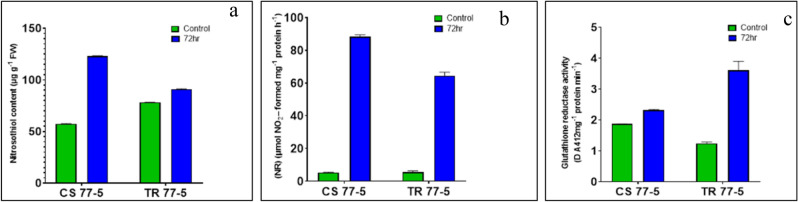
Effects of leaf rust pathogen on NO production Nitrosothiol accumulation (**a**) Nitrate reductase activity (**b**) and glutathione reductase activity (**c**) activity in leaves of wheat seedlings. Wheat genotypes Chinese spring (CS) (susceptible) and transfer (TR) (resistant line) inoculated with leaf rust pathotype 77-5. Samples were collected at 72 h after inoculation (HAI) and from uninoculated plants (C). Values are means (± SE) of 3 biological replicates. Graphs were created in Graph Pad prism 8 (www.graphpad.com).

To confirm NO's role further, the content of SNO and activity of key enzymes, NR and GR, were also analyzed (Fig. 9). The present result showed that incompatible interaction with pathotype 77–5 resulted in lower NR activity and nitric oxide burst strong enough for resistance. Nitrosothiol content was also significantly lower in incompatible interaction in comparison to compatible interaction. Further, resistance reaction promoted the degradation of GSSG by increasing the activity of GR, thereby increasing the content of GSH, thus improving the antioxidant capacity of the plant.

## Discussion

Leaf rust caused by *Puccinia triticina* Eriks. is ubiquitous and prevalent in occurrence in wheat-growing areas worldwide^2^. The genome-wide expression of *NIA* and *GSNOR* genes in responses to leaf rust infection was analyzed in the current study. Nitrate reductase/NIA is the major contributor to NO production in many higher plants like potato^50^ and rice^8^. Nitric oxide and the NO-derived molecules are called reactive nitrogen species (RNS) and include S-nitrosothiols (SNO) formed by NO's interaction with sulfhydryl-containing molecules like cysteine. S-nitrosothiols are of specific interest as they are stable in solution than NO and can help transport, store, and deliver NO to required locations, thus contributing to post-translational changes^11^. Amidst the several SNOs, S-nitroso glutathione (GSNO), a nitrosylation product, is most significant physiologically because of its function as a mobile NO reservoir. GSNORs catalyzes the NADH-dependent reduction of GSNO to GSSG and NH_3_. GSNOR activity can change the transnitrosation equilibrium between GSNO and S-nitrosylated proteins and, as a result, participates in the cellular NO homeostasis. Additionally, because this reaction affects the balance of GSH and NADH, GSNOR could be indirectly involved in the cellular redox states. By modulating the level of cellular SNO formation and homeostasis, GSNOR appears to regulate multiple forms of plant disease defence strategies^11^.

Nitrate reductase is one of the critical cytosolic enzymes involved in the production of NO during stress conditions^15^. In the case of *Arabidopsis,* two genes were identified which code for NR those are *Nia1* and *Nia2,* both located on chromosome 1^51^, and four NR genes in rice were annotated by The Rice Genome Annotation Project (RGAP) in 2018. We performed a BLASTP search using the *Arabidopsis Nia1* and *Nia2* and rice NIA protein’s amino acid sequence against the wheat genome available at Ensembl plants. We identified nine loci with an E-value of < 10^−10^ and identity > 50%. At different chromosome locations like Chr. 4A, Chr. 6A, Chr. 6B, Chr. 6D, Chr. 7A, and Chr. 7D. The NIA genes on the short and long arm of chromosome 6A are conserved across all the genome. This is a frequent phenomenon in wheat because of homoeology of three genomes A, B and D and conserved sequences required for major metabolic processes. The presence of homoeologs genes on all the genomes could also be due to their presence in diploid progenitor before evolution of hexaploid wheat^52^. While the other conditions where the homoeologs gene is missing in one or another genome could happen because of genome rearrangement during the process of polyploidization or further during evolution due to deletion.

S-nitrosoglutathione reductase, a class-III alcohol dehydrogenase that tightly controls intracellular GSNO levels by reducing GSNO to oxidized glutathione (GSSG) and ammonia (NH_3_) and thereby regulate the NO homeostasis^53^. *GSNOR* is a low copy number gene, and GSNOR proteins are rich in cysteine. Most of the higher plants have single copy numbers like *Arabidopsis*^54^. In the case of rice, also single *GSNOR* gene was annotated by The Rice Annotation Project (RAP) in 2018. While because of the allohexaploid nature of wheat, the gene is present on the long arm of all three homoeologs of Chr 6, suggesting that they are conserved across the progenitor genome of wheat before polyploidization.

The intracellular localization of identified *TaNIA *and *TaGSNOR* genes were predicted, many researchers determined the intracellular localization of NIA proteins. In maize leaf, it was found in cytoplasm^55^, and spinach (*Spinacia oleracea*) leaves it was found in chloroplasts^56^, so intracellular localization of NIA proteins depends on species. Wheat NIA proteins are located in the cytoplasm, chloroplast, and nucleus, according to our findings. GSNOR protein location, however, is conserved across species, i.e. in the cytoplasm^55^. GSNOR proteins were also found in the cytoplasm in our research.

In this study, we predicted the physiochemical properties including theoretical PI, molecular weight, instability index, aliphatic index, and grand average of hydropathicity (GRAVY) of TaNIA and TaGSNOR proteins. pH-related characteristics of a protein depend upon theoretical PI. At this point, protein has no charge and is less soluble, which facilitates protein isolation. The stability of a protein is inferred from the instability index. A value less than 40 signifies that protein is stable, while a value greater than 40 indicates that protein is unstable. In our study, three TaNIA proteins showed instability index value less than 40 and regular while the rest 6 TaNIA proteins have more than 40, indicating the protein's unstable nature. All the 3 TaGSNOR have an instability index of less than 40, showing all are stable proteins. The aliphatic index is the relative volume occupied by side aliphatic amino acid residues that signify the thermostability of protein^57^. All TaNIA and TaGSNOR proteins have a very high value of the aliphatic index that indicates higher thermostability. GRAVY is the measure of hydrophobicity or hydrophilicity^58^. A negative value specifies hydrophobic nature, while a positive value specifies the hydrophilic nature of a protein. In our case, all 9 TaNIA and 3 TaGSNOR proteins were hydrophobic.

Phylogeny analysis revealed the identification of two groups for TaNIA genes along with two Arabidopsis genes (*AtNIA1* and *AtNIA2*), one maize gene (*ZmNR*), and one rice NIA gene *OsNIA1* (Fig. 2a). On the other hand all the TaGSNOR genes were clustered with *GSNOR* genes from Arabidopsis and rice (*AtGSNOR* and *OsGSNOR*). Genomic and CDS sequences were retrieved and used to analyze gene structure that indicates the number of exons ranges for TANIAs from 2 to 3 while introns range from 1 to 2, while for all *TaGSNOR*s contain nine exons and eight introns. Conserved motif analysis revealed that TaNIA proteins contain 15 conserved regulatory motifs and TaGSNOR proteins contain eight conserved regulatory motifs. Homology modelling and 3-D structure analysis showed that all 9 TaNIA proteins and 3 TaGSNOR proteins exist as a homodimer. Although most of the proteins in wheat are still uncharacterized protein–protein interaction analysis. Based on observed co-expression of homologs in other species indicated the co-regulation of genes associated with nitrite assimilation, reductant supply etc.

miRNAs post transcriptionally regulate expression of target genes cleavage of target mRNA or by translational inhibition^59,60^. A number of studies has been conducted to observe the differential expression pattern of miRNAs during defence responses. A miRNA, tae-miR1136 targeting a cysteine-rich receptor-like protein kinase 41 showed two fold downregulation in response to stripe rust^60^. Similarly, miRNA, tae-miR1137b-5p, found downregulated during resistance reaction in stripe rust infected Louise (spring wheat cultivar)^61^. On the other hand, miRNA tae-miR1137a showed upregulation in response to powdery mildew *Bgt* and dysregulated expression to *Pst*, stripe rust ^62^. The above three described miRNAs were also identified as a putatively targets for two different NIA genes. The miRNA tae-miR1137b-5p and tae-miR1137a targets *TaNIA4-6b *while miR1136 targets *TaNIA9-7d*. The lower level of relative fold change in expression of target genes i.e. *TaNIA4-6b* and *TaNIA9-7d* may be due to these putative miRNAs expression.

In contrast, the TaGSNOR proteins interact with s-formyl glutathione hydrolase and aldehyde dehydrogenase. The spatial and temporal expression pattern of genes indicates their potential role in the development and stress responses. We analyzed the *in-silico* expression potential using exVIP. Expression of three TaNIA genes (*TaNIA2-6a*, *TaNIA4-6d*, and *TaNIA6-6b*) showed a visible increase after 48 h and 72 h of stripe rust infection. Similarly, *TaGSNOR* genes (*TaGSNOR2* and *TaGSNOR3*) showed upregulation after 48 h and 72 h of powdery mildew and stripe rust infection.

To further validate and analyze *TaNIA* genes and *TaGSNOR* genes in response to leaf rust infection in wheat by quantitative RT-PCR (qRT-PCR) analysis, experiments were conducted in the seedling stage in two wheat genotypes CS and TR. Out of the 9 TaNIAs, 8 TaNIAs expression analysis was performed except TaNIA5. In response to leaf rust inoculation, expression of all TaNIA genes was up-regulated in the incompatible reaction compared to the compatible reaction, inferring the role of TaNIA genes in disease resistance. Similar results were obtained by Qiao et al.^49^. They found NO production increases at 24 HAI and 72 HAI when wheat cultivar Lovrin 10 was infected with leaf rust. This result is an indirect measure as NO is produced mainly by nitrate reductase. *AtNIA1* and *AtNIA2* control NO production during the resistance to pathogen invasions^63^ in *Arabidopsis*. Recently Lu et al.^9^ also reported the involvement of *OsNIA1* in resistance to RBSDV infection partially through a salicylic acid-dependent pathway. In comparison, wild-type cultivar *Osnia2* mutant rice plants accumulated lower levels of NO after RBSDV infection^9^. These genetic findings thus support our results, indicating that NO production by the NR pathway might be playing an essential role in wheat resistance to leaf rust infection.

Nitric oxide reacts with reduced glutathione (GSH) in the presence of O_2_ to form S-nitrosoglutathione (GSNO). As explained previously, GSNOR regulates the cellular level of SNPs and therefore determines the cellular GSNO content. Mutation of *Arabidopsis thaliana AtGSNOR1* or the antisense suppression of GSNOR resulted in resistance to *Peronospora parasitica*, seemingly connected with higher levels of intracellular SNOs^64^. Whereas in a *Plasmopara halstedii* resistant sunflower (*Helianthus annuus* L.) cultivar, the reverse was true^19^ in the case of infected hypocotyls between GSNOR activity and GSNO distribution and content. Chaki et al.^19^ found that in sunflower-mildew interaction, GSNO accumulated in the cortex cells. After the interaction with the pathogen, GSNO was relocated to the epidermis, the site of infection of the pathogen. The redistribution of GSNO after pathogen interaction assists resistance reaction in sunflower-mildew interaction. Among 3 *TaGSNOR* genes, only *TaGSNOR2* was highly up-regulated by leaf rust pathogen at 24 and 72 HAI, followed by a sharp decrease in expression at 144 HAI. Expression of *TaGSNOR3* has up-regulated both incompatible and incompatible interaction at 24HAI followed by down-regulation indicates its likely involvement in basal response/pattern triggered immunity. As demonstrated by Feechan et al.^65^ that GSNOR has a role in basal resistance.

Under the catalysis of Glutathione reductase (GR), the oxidized form of glutathione (GSSG) can be readily converted to the reduced state (GSH). GSH is a major intracellular antioxidant that eliminates reactive oxygen species (ROS). GR maintains a high GSH/GSSG ratio by reducing GSSG to play a key role in the antioxidant defence process. In support of the expression analysis, we performed NO visualization and biochemical analysis of nitrosothiol, nitrate reductase, and glutathione reductase. Based on previous reports^49^, 72 hAI was selected to study NO localization. The 72  HAI leaf samples were taken for NO visualization. NO was visible on the guard cells on both compatible and incompatible combinations. NO fluorescence gradually intensified in the mesophyll cell around the infected site. However, no lesions were formed in incompatible interaction, and NO fluorescence was limited to spherical structures suspected to be fungal spores. Guo et al.,^66^ demonstrated that NO accumulation was there in early-stage and in the later stage in incompatible reactions.

In contrast, the NO accumulation was found only in later stages incompatible reaction. Upregulation of *TaNI*A genes and visualization of NO on leaf rust inoculated leaf showed that *TaNIA* is one of the primary sources of NO in wheat in response to leaf rust. Our result reveals that incompatible interaction with leaf rust pathogen resulted in high NR activity and nitric oxide burst intense enough for resistance and no disease lesion development. Nitrosothiol content was also significantly lower in incompatible interaction in comparison to compatible interaction. It can be correlated with the increase in GR activity in incompatible interaction as GR tends to maintain the homeostasis of the NO in vivo. Resistance reaction also promoted the degradation of GSSG by increasing the activity of GR, thereby increasing the content of GSH, thus improving the antioxidant capacity of the plant.

## Conclusions

The result showed that incompatible interaction resulted in high NR activity and NO burst strong enough to resist disease development. Nitrosothiol content was also significantly lower in incompatible interaction in comparison to compatible interaction. Resistance reaction also promoted the degradation of GSSG by increasing the activity of GR, thereby increasing the content of GSH, thus improving the antioxidant capacity of the plant. Delledonne et al.,^67^ reported that NO could potentiate hypersensitive cell death concomitant with the ROS burst during inoculation with a virulent pathogen. Still, less ROS were produced together with NO in response to a virulent strain. In our study, also NO alleviated oxidative stress (data not shown) and during incompatible interaction with leaf rust. Again, these results indicate that different mechanisms underlie NO-modulated resistance responses between compatible and incompatible interaction with leaf rust. The data presented in the study suggests the involvement of NR-mediated NO production and expression of NIA and GSNOR transcripts in regulating leaf rust resistance and upregulation of antioxidant enzyme activities. The activity of GR further supports that NO generation and increased antioxidant enzyme activities are required for maintaining the redox state and protection against leaf rust in wheat.

## Supplementary Information

Supplementary Tables.

Supplementary Figures.
