# Supplementary material for: Analysis of NIA and GSNOR family genes and nitric oxide homeostasis in response to wheat-leaf rust interaction
Source: Sci Rep. 2022 Jan 17;12:803. doi: 10.1038/s41598-021-04696-5 (PMC8764060; doi:10.1038/s41598-021-04696-5)
Supplement: Supplementary file 1 — Supplementary Tables. [file 41598_2021_4696_MOESM1_ESM.docx]

**Supplementary Table 1:** Gene specific primers used for qRT-PCR analysis of TaNIA and TaGSNOR genes

| **S.No** | **Gene** | **Primer** | **Sequence** | **Length** |
| --- | --- | --- | --- | --- |
| 1 | TaNIA1 | F | CATGGGAGTGCAGCCTGTT | 19 |
|  |  | R | GCAAGCTACTCGTTGAAGATT | 21 |
| 2 | TaNIA2 | F | CCTTCATTCACACCACCGA | 19 |
|  |  | R | GCGTTGATGAGGATGCTGT | 19 |
| 3 | TaNIA3 | F | AAGAAGGAGCTCTCCCACG | 19 |
|  |  | R | CATGCTCGTCGGAGTGTAGG | 20 |
| 4 | TaNIA4 | F | CCTTCCTCAAGGACCATCC | 19 |
|  |  | R | CAGAGTTATAGCCAGCGCCA | 20 |
| 5 | TaNIA5 | F | GCTACTACCACTACCGCGA | 19 |
|  |  | R | TGATCATGCACTCCGGCTT | 19 |
| 6 | TaNIA6 | F | TCTGCACGACTTCTCCTCC | 19 |
|  |  | R | GCCATGCTGTGTTTGTGTT | 19 |
| 7 | TaNIA7 | F | TTGGATGCTATGCACGCTG | 19 |
|  |  | R | CTTCTCTGGCTGGGTGTTG | 19 |
| 8 | TaNIA8 | F | ATACGCGAACCGAACGGAG | 19 |
|  |  | R | CTGAACTTCCAGCCTTCCT | 19 |
| 9 | TaNIA9 | F | CGTGGGTAGGGTCAATGAA | 19 |
|  |  | R | CCTGCGCTATCCGCTTAGAA | 20 |
| 10 | TaGSNOR1 | F | GAACACGAACCGCTGCTAT | 19 |
|  |  | F | AAGTTGCCGTTGCAAGCAC | 19 |
| 11 | TaGSNOR2 | R | TGTTGAGAGTGTCGGCGAA | 19 |
|  |  | F | CCCATTAACCGAGAAGCGG | 19 |
| 12 | TaGSNOR3 | R | ACTCTTGAACACGAACCGCT | 20 |
|  |  | F | TTGCCGTTGCAAGCACATA | 19 |

**Supplementary Table 2:** Putative cis-elements of Wheat *TaTrx* genes identified using PlantCARE database

| **Gene** | **CARE** | **Sequence** | **Start** | **Length** | **Strand** | **Function** |
| --- | --- | --- | --- | --- | --- | --- |
| TaNIA1 | TC-rich repeats | GTTTTCTTAC | 3587 | 9 | - | CARE involved in defense and stress responsiveness |
| TaNIA2 | TC-rich repeats | ATTCTCTAAC | 2886 | 9 | - | CARE involved in defense and stress responsiveness |
| TaNIA2 | TC-rich repeats | GTTTTCTTAC | 3236 | 9 | + | CARE involved in defense and stress responsiveness |
| TaNIA2 | TC-rich repeats | GTTTTCTTAC | 4540 | 9 | - | CARE involved in defense and stress responsiveness |
| TaNIA4 | WUN-motif | AAATTACTA | 4665 | 9 | + | CARE involved in defense and stress responsiveness |
| TaNIA6 | TC-rich repeats | GTTTTCTTAC | 3115 | 9 | + | CARE involved in defense and stress responsiveness |
| TaNIA6 | TC-rich repeats | ATTCTCTAAC | 5288 | 9 | + | CARE involved in defense and stress responsiveness |
| TaNIA9 | TC-rich repeats | GTTTTCTTAC | 3890 | 9 | - | CARE involved in defense and stress responsiveness |

**Supplementary Table 3:** Details of predicted gene-specific SSR motifs for all the identified genes.

| **Gene** | **SSR** | **Forward primer(5'-3')** | **Tm** | **Reverse primer(5'-3')** | **Tm** | **product** |
| --- | --- | --- | --- | --- | --- | --- |
| ***TaNR/NIA*** | | | | | | |
| TaNIA1-4a | (CGG)5 | ATCATGGGCCTCAACAAG | 56.89 | CGTAGGCGAGCATGATGT | 58.32 | 151 |
| TaNIA2-6a | (CGG)4 | ATCATGTCCTCCAAAAAGG | 54.93 | GTAGGCGAGCAGGATGTC | 56.78 | 144 |
| TaNIA3-6a | (CTC)4 | GAGTACAACCGTCAGGTGTC | 55.48 | GTCCTCGTCGTCCGAATC | 59.13 | 141 |
| TaNIA4-6b | (CGG)4 | CTGAACGTGTGCTTCGAG | 56.42 | AGTGGTAGTAGTTGTCGGACTC | 55.09 | 256 |
| TaNIA5-6b | (CGG)4 | AAGGAGCAGAACATGGTG | 54.38 | CATGTAGGCGAGGATGAC | 54.81 | 252 |
| TaNIA6-6d | (AT)7 | GATCGAAGATAGAAGCTTGGT | 55.3 | TCCTACGTGAGCATAAAACAT | 55.02 | 166 |
| TaNIA7-6d | (CTC)4 | GTCTGCAACCACTTAACAATG | 55.84 | GTCCTCGTCGTCCGAGTC | 59.31 | 165 |
| TaNIA8-7a | (CGG)5 | AAGGAGCAGAACATGGTG | 54.38 | CATGATGTCCCTGGAAGG | 57.23 | 246 |
| TaNIA9-7d | (CGG)6 | ATCATGGGCCTCAGGAAG | 58.53 | GAGCATGATGTCTCTGGAAG | 55.81 | 147 |
|  |  |  |  |  |  |  |

**Supplementary Table 4:** List of identified miRNAs targeting wheat TaNIAs.

| **miRNA_Acc.** | **Target_Acc.** | **miRNA_Acc.** | **Target_Acc.** |
| --- | --- | --- | --- |
| ***TaNR/NIA*** |  | ***TaGSNOR*** |  |
| tae-miR1137b-5p | TaNIA4-6b | tae-miR1139 | TaGSNOR1 |
| tae-miR1137a | TaNIA4-6b | tae-miR1131 | TaGSNOR3 |
| tae-miR1133 | TaNIA4-6b | tae-miR1131 | TaGSNOR2 |
| tae-miR531 | TaNIA2-6a | tae-miR1131 | TaGSNOR1 |
| tae-miR1127a | TaNIA4-6b | tae-miR9663-5p | TaGSNOR3 |
| tae-miR1136 | TaNIA9-7d |  |  |
| tae-miR398 | TaNIA5-6b |  |  |
| tae-miR5384-3p | TaNIA7-6d |  |  |
| tae-miR9657a-3p | TaNIA9-7d |  |  |
| tae-miR9657a-3p | TaNIA8-7a |  |  |
| tae-miR9670-3p | TaNIA2-6a |  |  |
| tae-miR1134 | TaNIA5-6b |  |  |
| tae-miR398 | TaNIA7-6d |  |  |
| tae-miR408 | TaNIA4-6b |  |  |
| tae-miR408 | TaNIA2-6a |  |  |
| tae-miR408 | TaNIA9-7d |  |  |
| tae-miR408 | TaNIA1-4a |  |  |
| tae-miR408 | TaNIA8-7a |  |  |
| tae-miR5384-3p | TaNIA7-6d |  |  |
| tae-miR5384-3p | TaNIA3-6a |  |  |
| tae-miR5384-3p | TaNIA5-6b |  |  |
| tae-miR9657a-3p | TaNIA9-7d |  |  |
| tae-miR9657b-3p | TaNIA9-7d |  |  |
| tae-miR9657c-3p | TaNIA9-7d |  |  |
| tae-miR9660-5p | TaNIA7-6d |  |  |
| tae-miR9676-5p | TaNIA2-6a |  |  |
| tae-miR9780 | TaNIA6-6d |  |  |
|  |  |  |  |
|  |  |  |  |

**Supplementary Table 5:** Homology modeling of wheat TaNIA and TAGSNOR proteins using automated Swiss-Model server, along with their Ramachandran favoured percentage.

| **S.No** | **Protein** | **Seq. Identity (%)** | **QMEAN** | **Template** | **Template Description** | **Oligo-state** | **Ramachandran favoured (%)** |
| --- | --- | --- | --- | --- | --- | --- | --- |
|  | ***TaNA/NIA*** |  |  |  |  |  |  |
| 1 | TaNIA1 | 44.77 | -1.42 | 2bih.1.A | Nitrate reductase [NADPH domain] | homo-dimer | 94.16 |
|  |  | 84.96 | -1.4 | 1cnf.1.A | Nitrate reductase [cytochrome b domain] | monomer | 91.12 |
| 2 | TaNIA2 | 45.12 | -1.6 | 2bih.1.A | Nitrate reductase [NADPH domain] | homo-dimer | 93.65 |
|  |  | 87.22 | -1.31 | 1cnf.1.A | Nitrate reductase [cytochrome b domain] | monomer | 88.76 |
| 3 | TaNIA3 | 45.26 | -2.26 | 2bih.1.A | Nitrate reductase [NADPH domain] | homo-dimer | 94.34 |
|  |  | 66.54 | -1.72 | 1cnf.1.A | Nitrate reductase [cytochrome b domain] | monomer | 89.58 |
| 4 | TaNIA4 | 44.53 | -1.15 | 2bih.1.A | Nitrate reductase [NADPH domain] | homo-dimer | 94.47 |
|  |  | 87.22 | -1.39 | 1cnf.1.A | Nitrate reductase [cytochrome b domain] | monomer | 88.76 |
| 5 | TaNIA5 | 45.5 | -2.11 | 2bih.1.A | Nitrate reductase [NADPH domain] | homo-dimer | 93.63 |
|  |  | 66.54 | -1.35 | 1cnf.1.A | Nitrate reductase [cytochrome b domain] | monomer | 93.82 |
| 6 | TaNIA6 | 44.88 | -1.94 | 2bih.1.A | Nitrate reductase [NADPH domain] | homo-dimer | 94.01 |
|  |  | 86.84 | -1.42 | 1cnf.1.A | Nitrate reductase [cytochrome b domain] | monomer | 88.76 |
| 7 | TaNIA7 | 45.5 | -1.97 | 2bih.1.A | Nitrate reductase [NADPH domain] | homo-dimer | 93.75 |
|  |  | 65.66 | -2 | 1cnf.1.A | Nitrate reductase [cytochrome b domain] | monomer | 89.58 |
| 8 | TaNIA8 | 45.01 | -1.33 | 2bih.1.A | Nitrate reductase [NADPH domain] | homo-dimer | 95.07 |
|  |  | 84.96 | -1.26 | 1cnf.1.A | Nitrate reductase [cytochrome b domain] | monomer | 90.35 |
| 9 | TaNIA9 | 44.77 | -1.45 | 2bih.1.A | Nitrate reductase [NADPH domain] | homo-dimer | 95.34 |
|  |  | 85.71 | -1.7 | 1cnf.1.A | Nitrate reductase [cytochrome b domain] | monomer | 88.8 |
|  | ***TaGSNOR*** |  |  |  |  |  |  |
| 1 | TaGSNOR 1 | 87.77 | 0.32 | 4dl9.1.A | Alcohol dehydrogenase class III | Homo-dimer | 96.79 |
| 2 | TaGSNOR 2 | 88.56 | 0.25 | 4dl9.1.A | Alcohol dehydrogenase class III | Homo-dimer | 96.79 |
| 3 | TaGSNOR 3 | 88.03 | 0.33 | 4dl9.1.A | Alcohol dehydrogenase class III | Homo-dimer | 96.79 |

**Supplementary Table 6**: Details of overlapping of *TaNIA* and *TaGSNOR* proteins

| **Protein** | **Structure overlap (%)** | **RMSD** | **Fragment score** | **Topology score** | **Match size** | **Identical residues** | **Identical residues with Heuristics** | **Z-Score** |
| --- | --- | --- | --- | --- | --- | --- | --- | --- |
| TaNIA1 and TaNIA3 | 98.47 | 0.36 | 1 | 1 | 839 | 645 | 645 | 20.52 |
| TaNIA2 and TaNIA4 | 99.77 | 0.19 | 1 | 1 | 852 | 820 | 820 | 21.02 |
| TaNIA2 and TaNIA6 | 99.41 | 0.2 | 1 | 1 | 849 | 822 | 822 | 20.93 |
| TaNIA3 and TaNIA7a | 100 | 0.09 | 1 | 1 | 852 | 842 | 842 | 21.15 |
| TaNIA3 and TaNIA7 | 100 | 0.09 | 1 | 1 | 852 | 842 | 842 | 21.15 |
| TaNIA3 and TaNIA5 | 100 | 0.08 | 1 | 1 | 852 | 848 | 848 | 21.15 |
| TaNIA8 and TaNIA9 | 99.07 | 0.37 | 1 | 1 | 848 | 814 | 814 | 20.59 |
| TaGSNOR1 and TaGSNOR2 | 99.73 | 0.65 | 1 | 0.5 | 743 | 730 | 730 | 20.4 |
| TaGSNOR1 and TaGSNOR3 | 99.73 | 0.65 | 1 | 0.5 | 743 | 734 | 734 | 20.4 |
