# Supplementary material for: Analysis of NIA and GSNOR family genes and nitric oxide homeostasis in response to wheat-leaf rust interaction
Source: Sci Rep. 2022 Jan 17;12:803. doi: 10.1038/s41598-021-04696-5 (PMC8764060; doi:10.1038/s41598-021-04696-5)
Supplement: Supplementary file 2 — Supplementary Figures. [file 41598_2021_4696_MOESM2_ESM.docx]

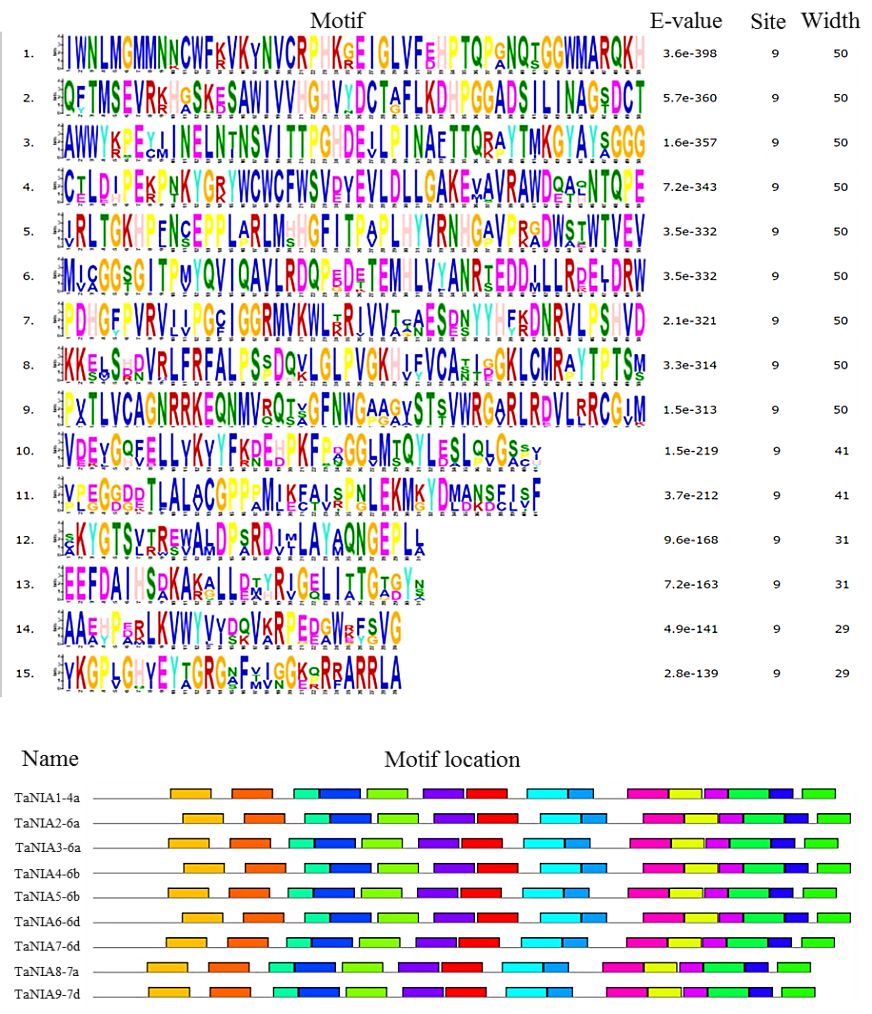


**Supplementary Figure 1 (a)** Identified regulatory Conserved motif for TaNIA proteins using MEME (b) Location of the motif on the TaNIA protiens. Image was created in MEME software version 5.0.5 ((<http://meme-suite.org/meme_5.0.5/>).

**b)**

**a)**


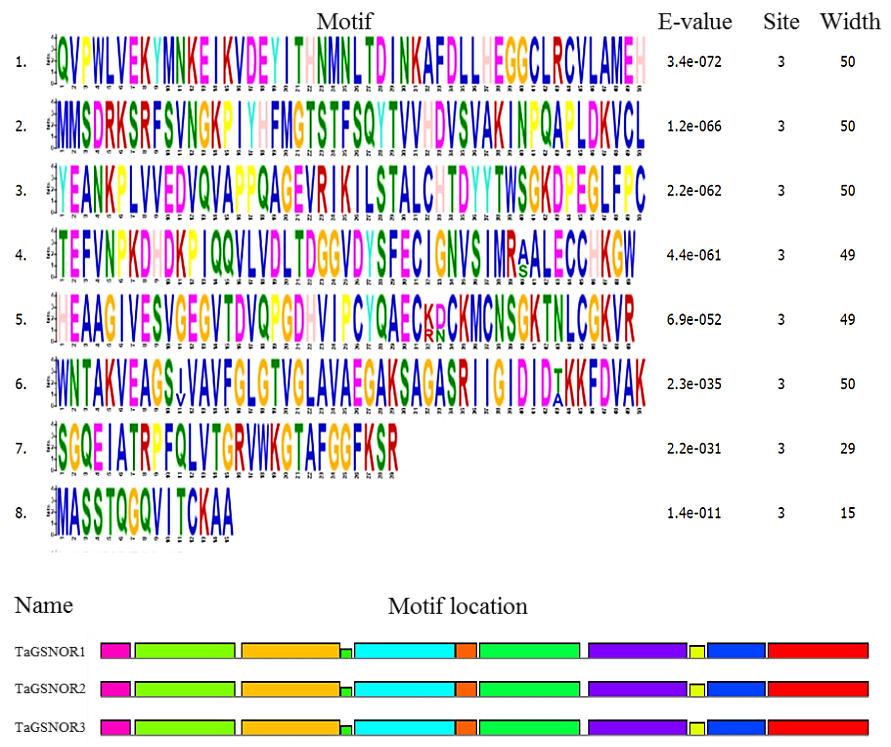


**a)**

**b)**

**Supplementary Figure 2: (a)** Identified regulatory Conserved motif for TaGSNOR proteins using MEME **(b)** Location of the motif on the TaGSNOR protiens. Image was created in MEME software version 5.0.5 ((http://meme-suite.org/meme_5.0.5/).

**Supplementary Fig. 3 Potential** miRNAs targeting *TaNIA* and *TaGSNOR* genes identified usin g psRNATarget server depicted as miRNA network model. Image was constructed using desktop application of cytoscape (Cytoscape 3 5 1)


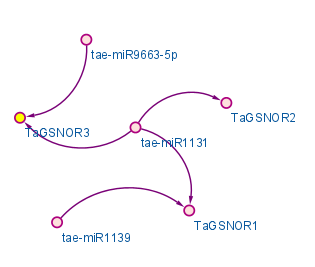

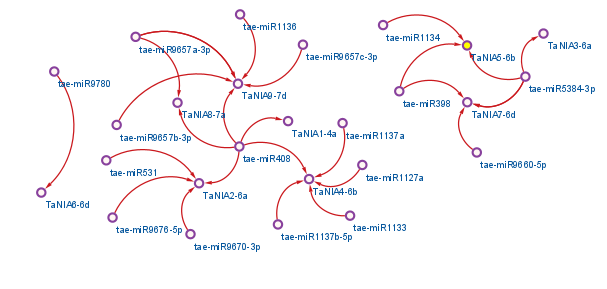
**Supplementary Figure 4 Identification of functional parters of TaNIA:** **Traes_6DS_69570DBE2.1 (a) and TAGSNOR;** Traes_6DL_FD8A6A45F.1 **(b) by coexpression analysis** in STRING tool. Images were created using STRING (<https://string-db.org/cgi/coexpression>).

**b)**

**a)**

| **(a)** | 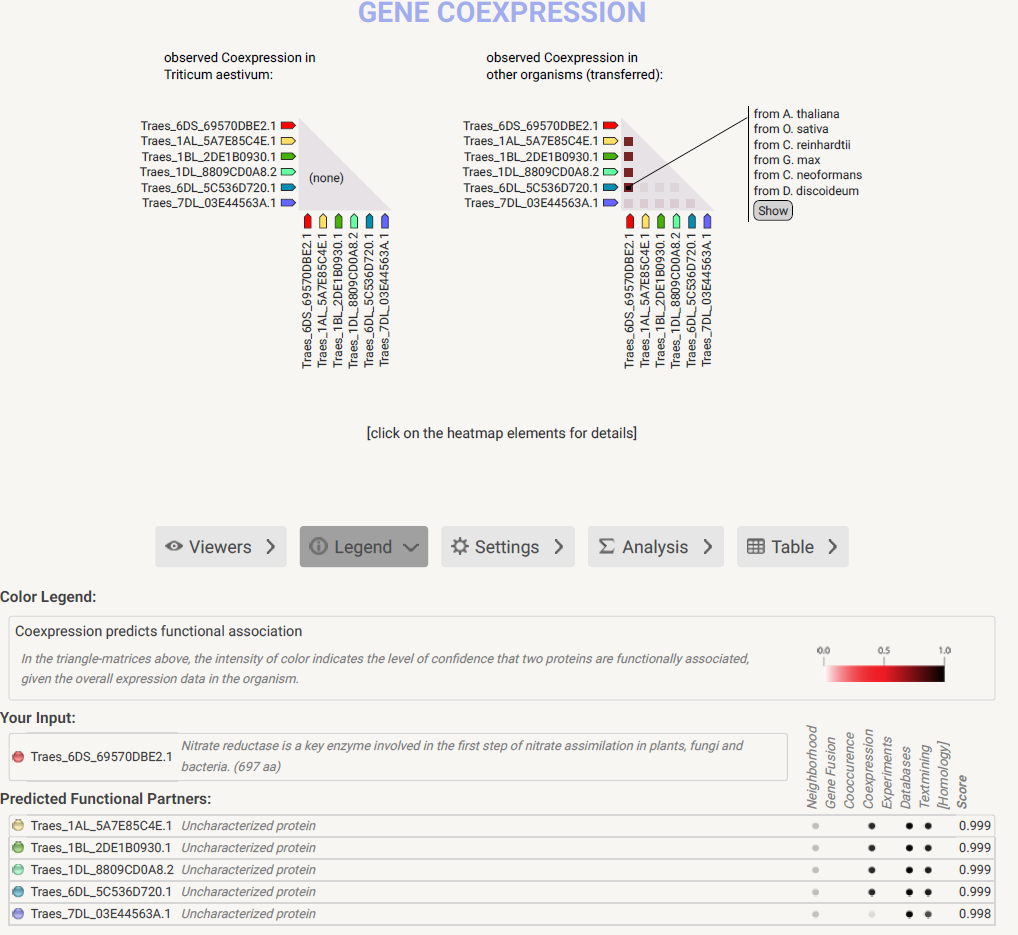 |
| --- | --- |
| (b) | 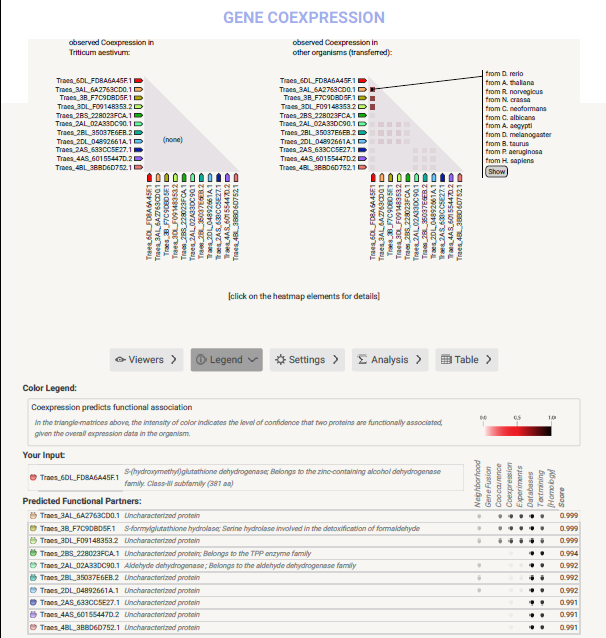 |


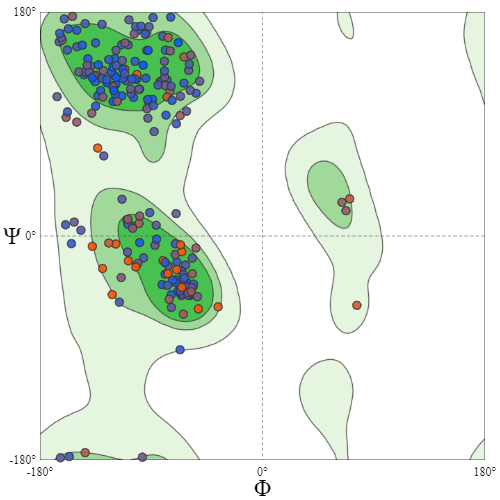

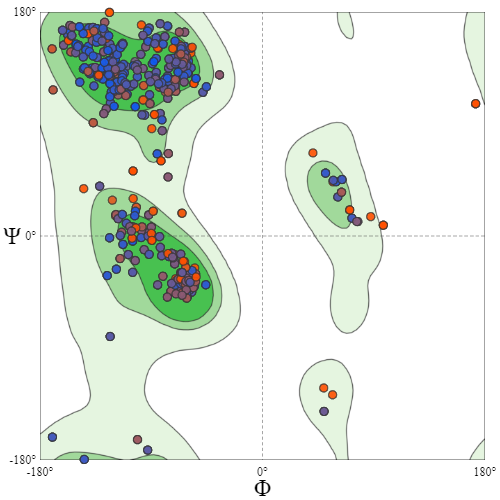

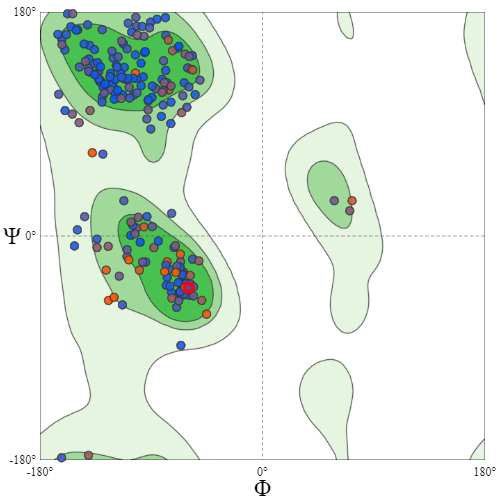

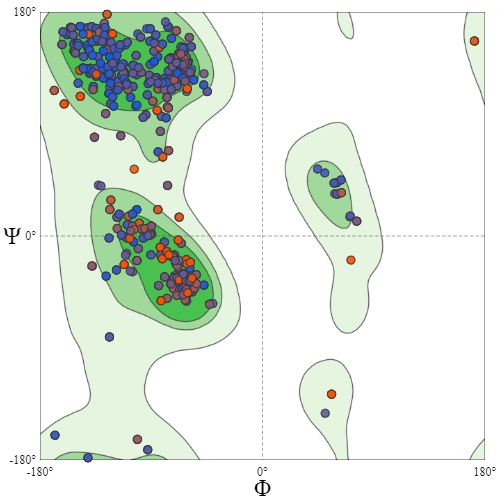

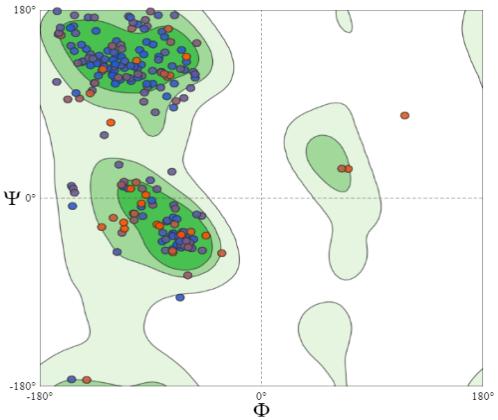

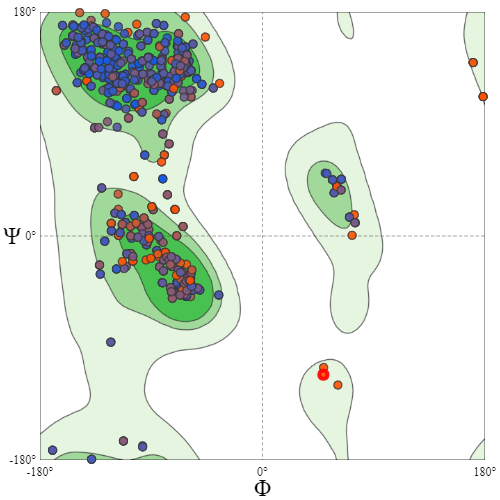

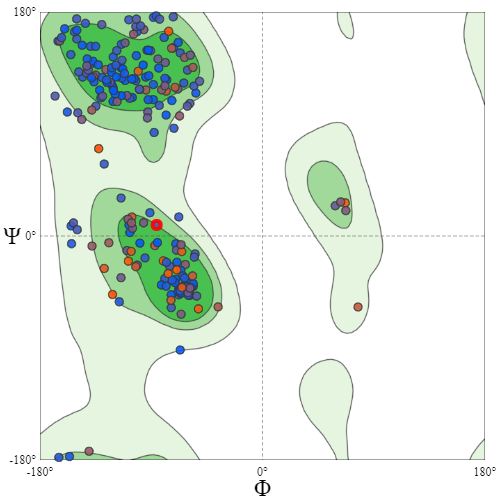

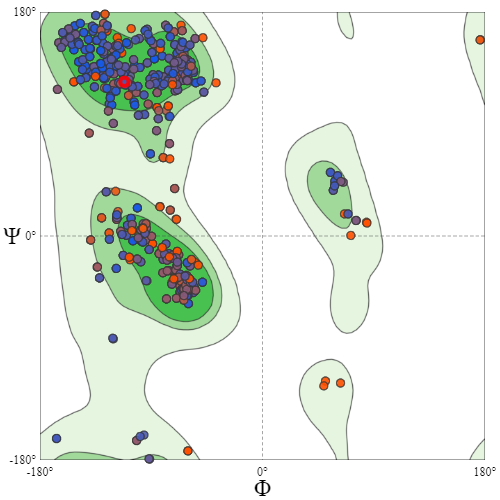

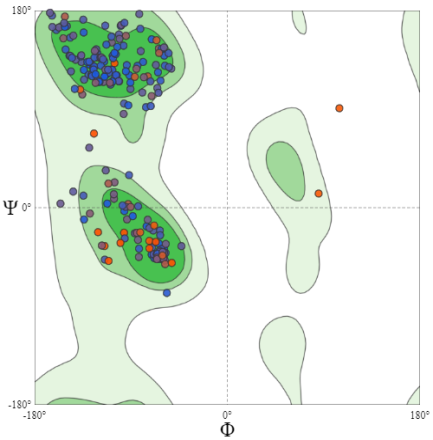

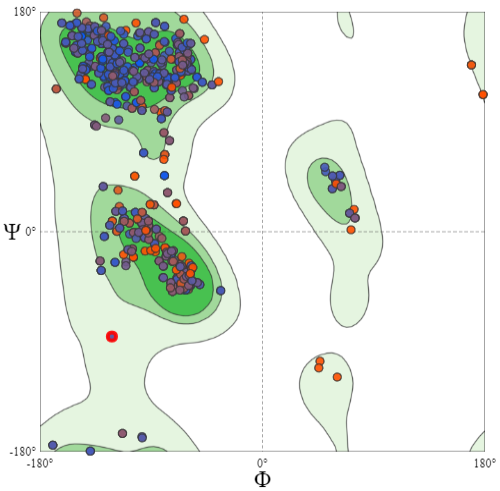

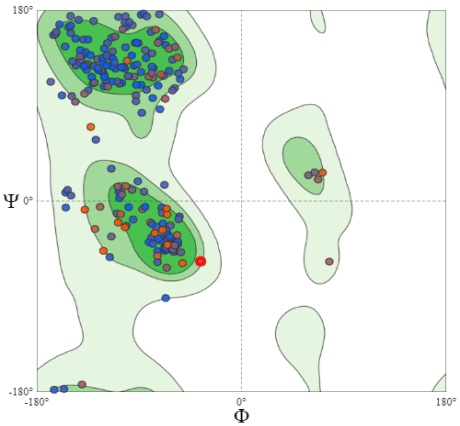

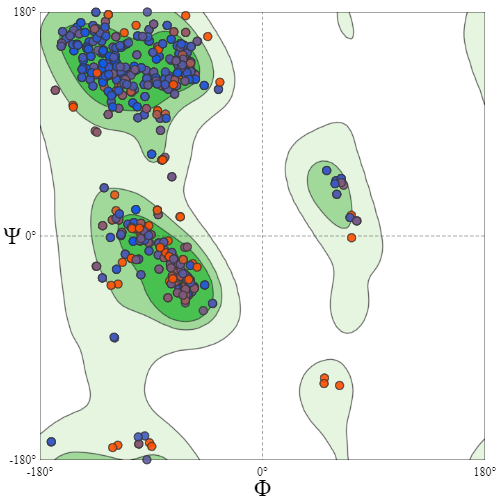

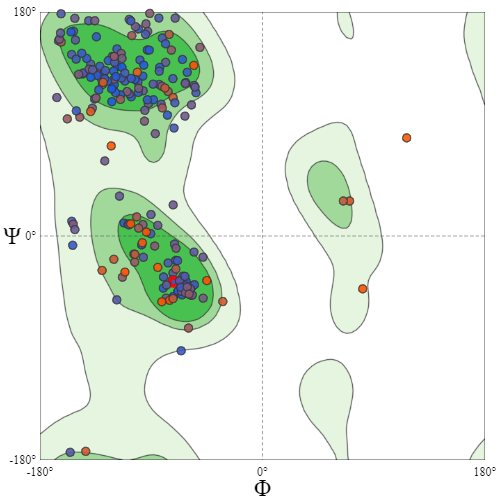

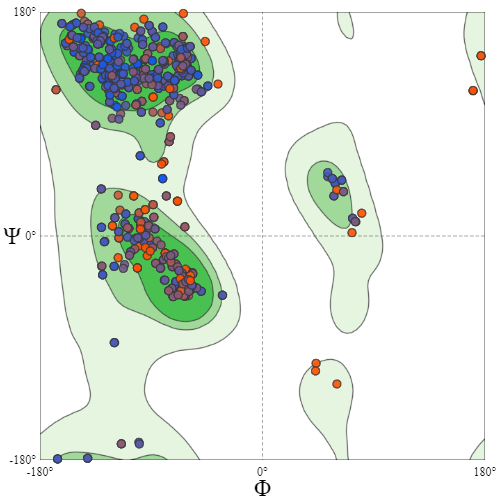

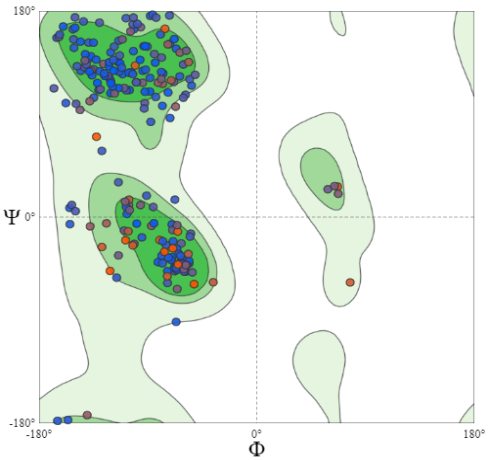

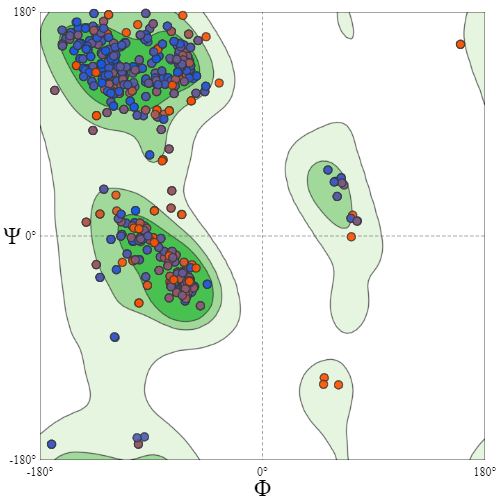

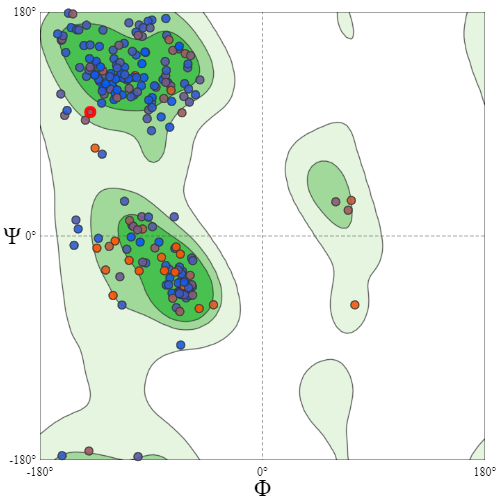

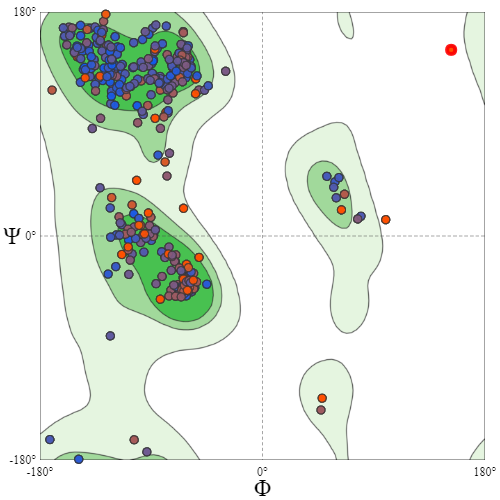

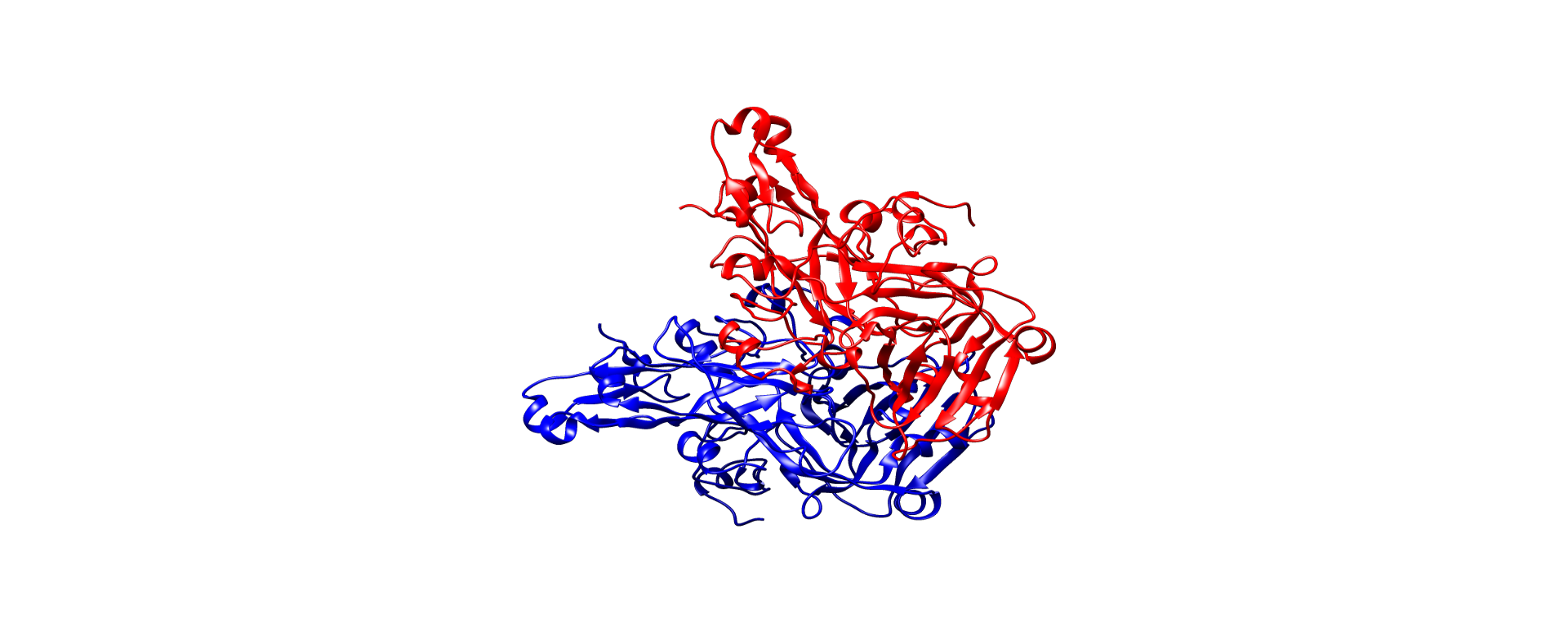

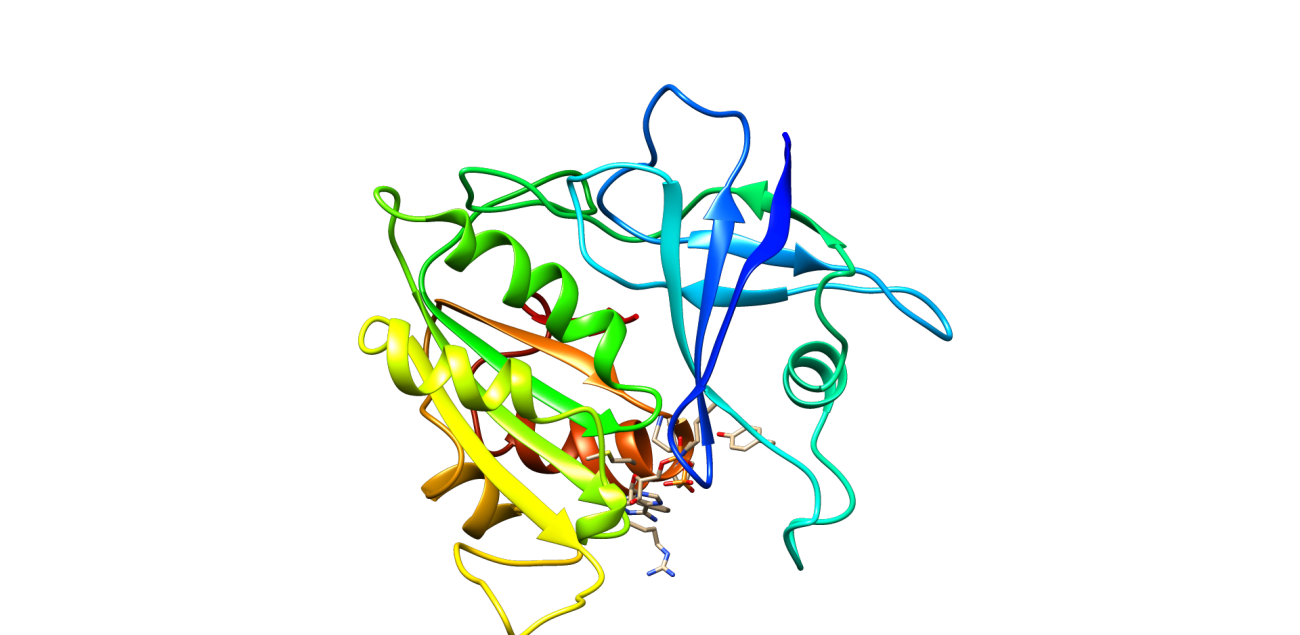

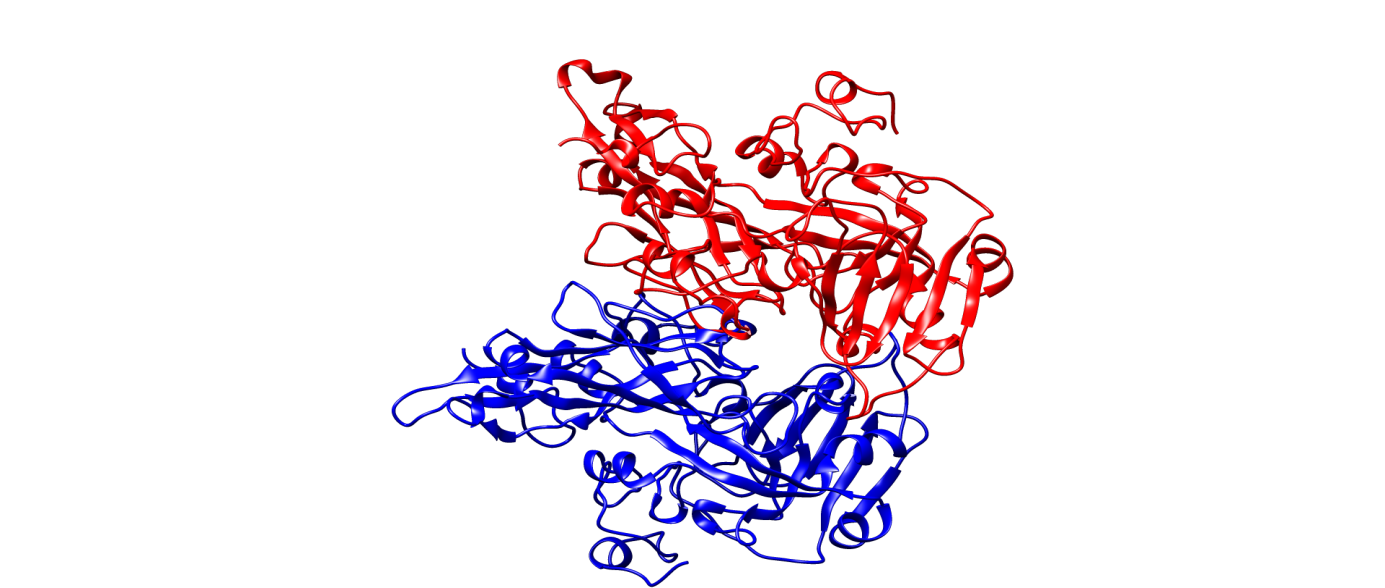

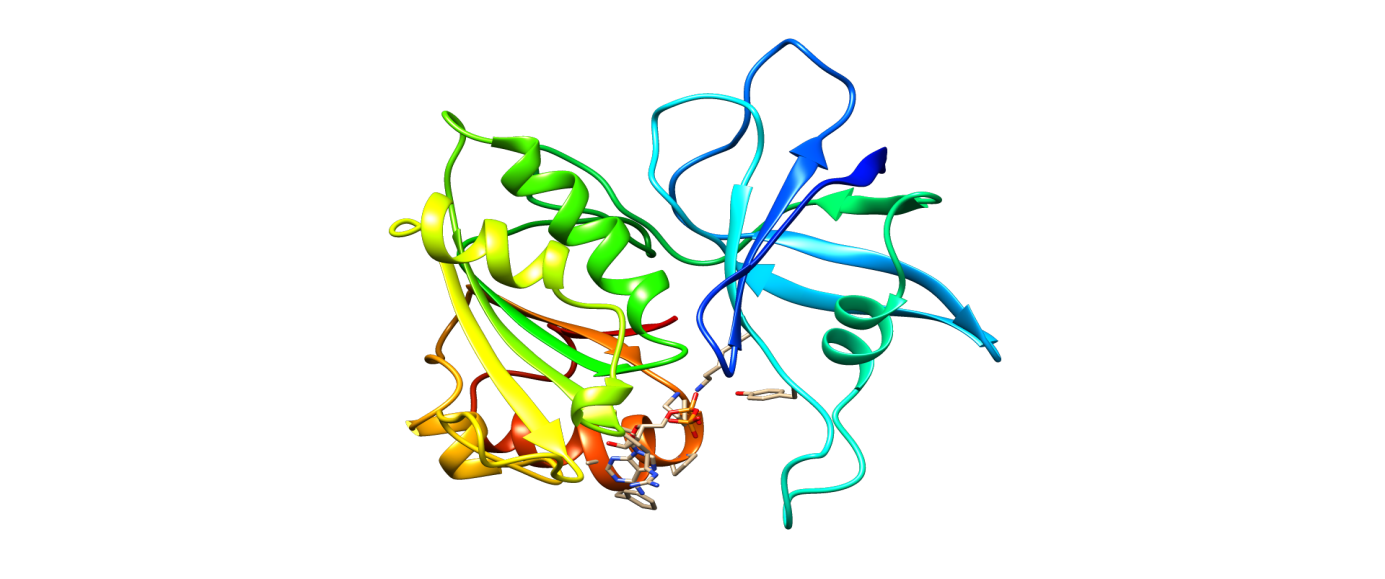

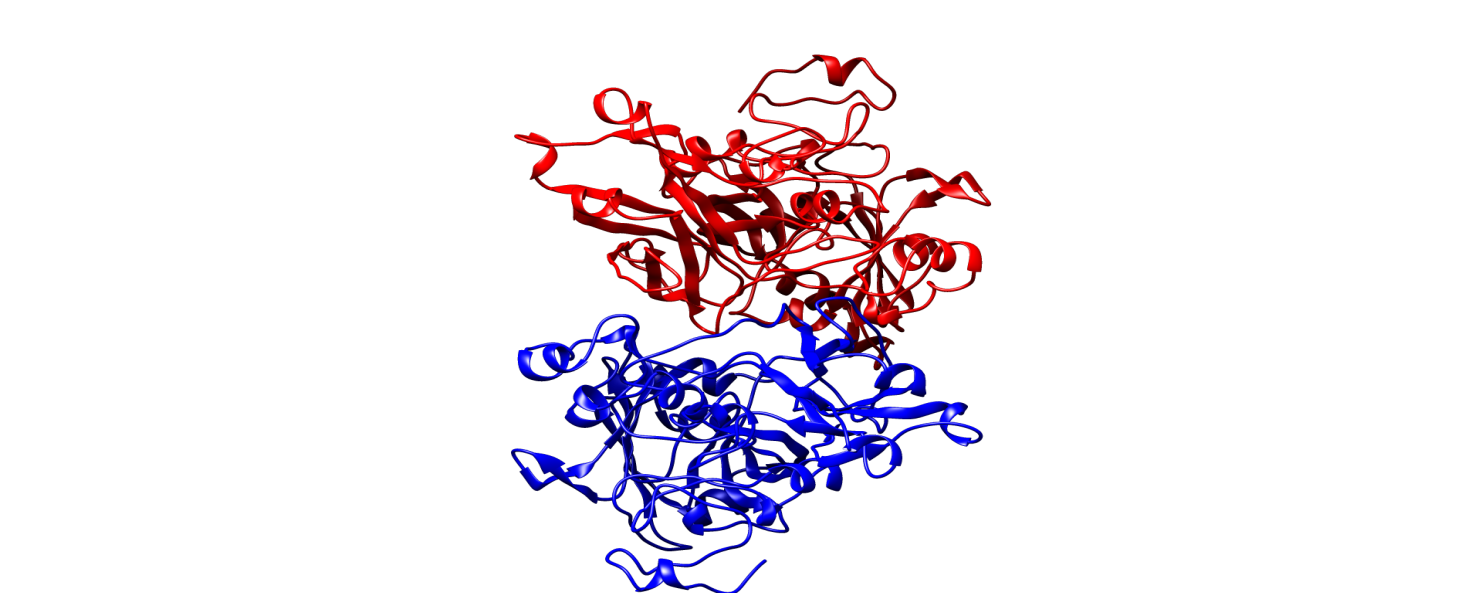

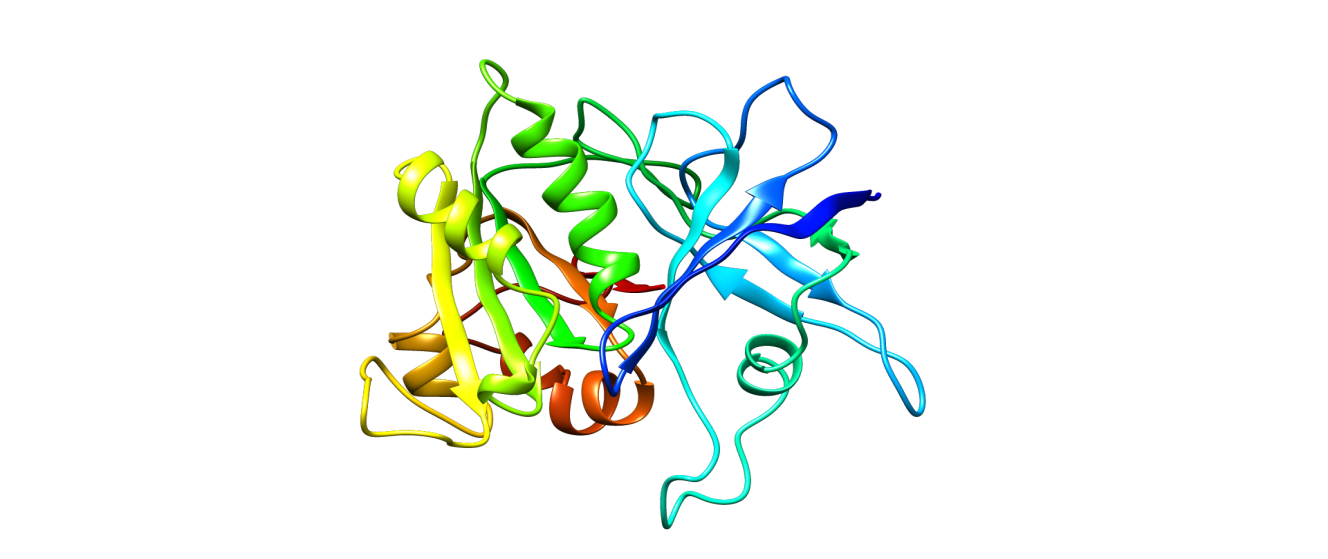

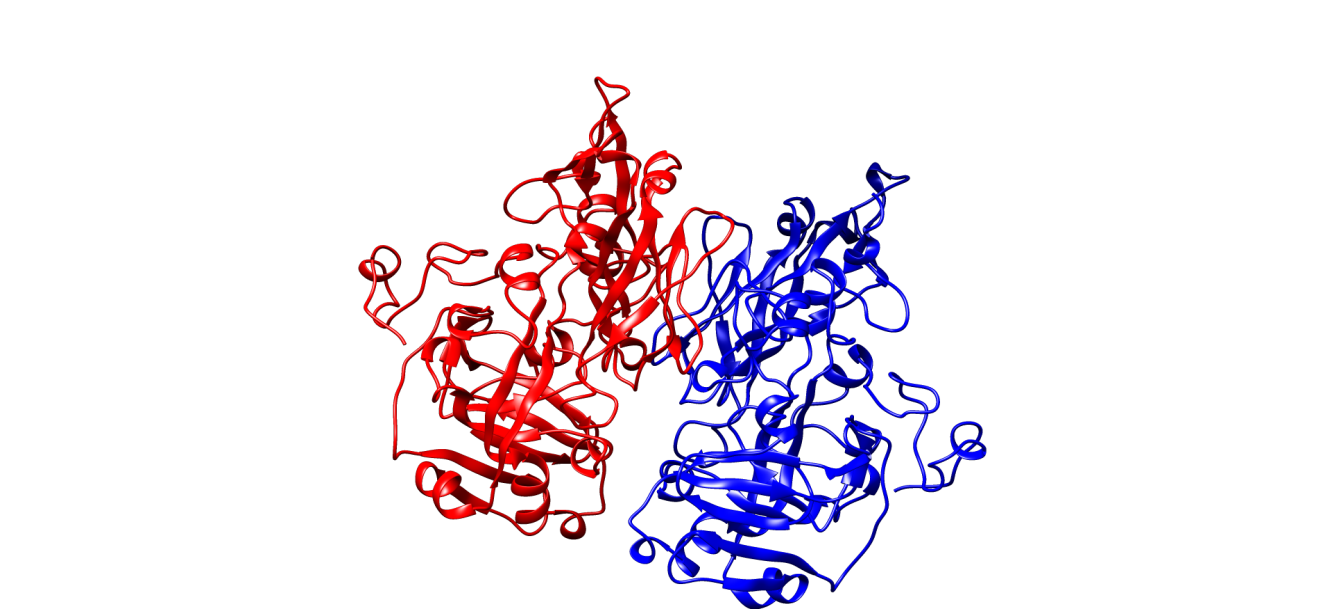

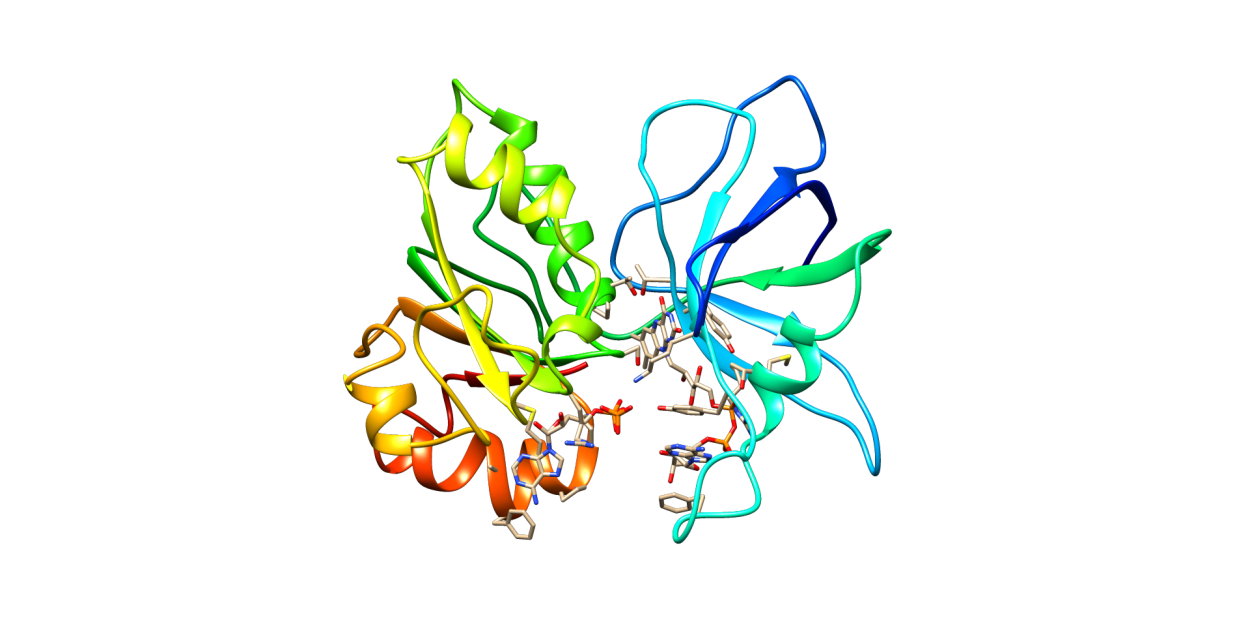

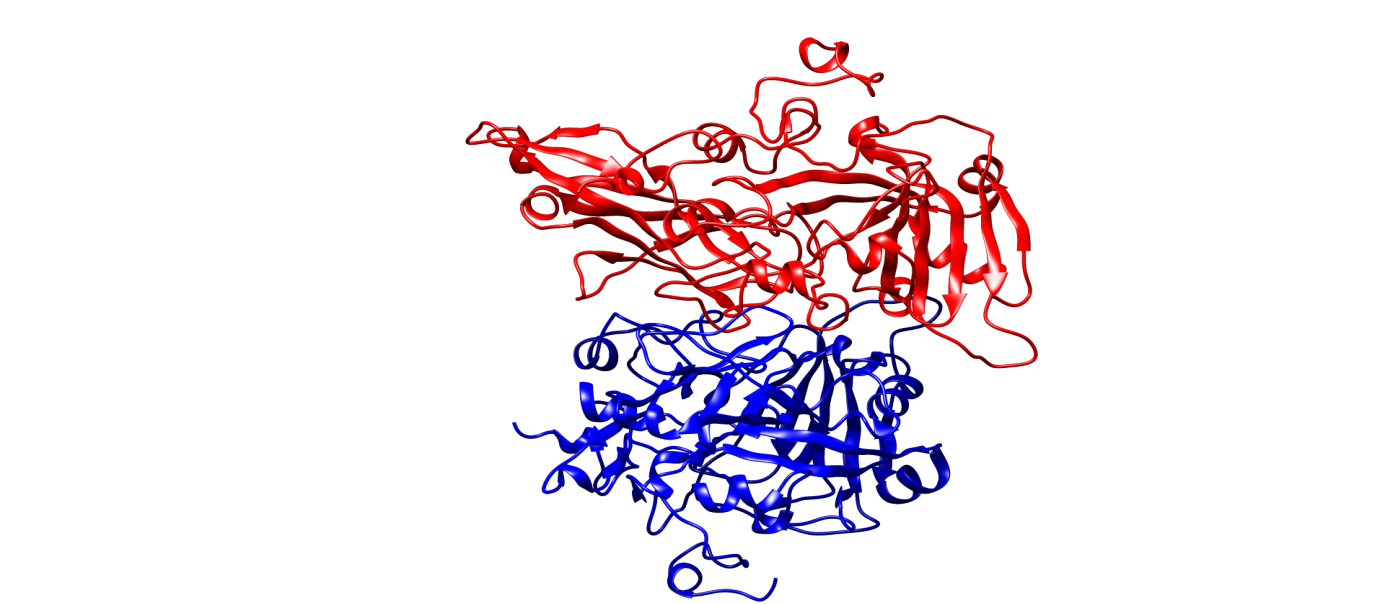

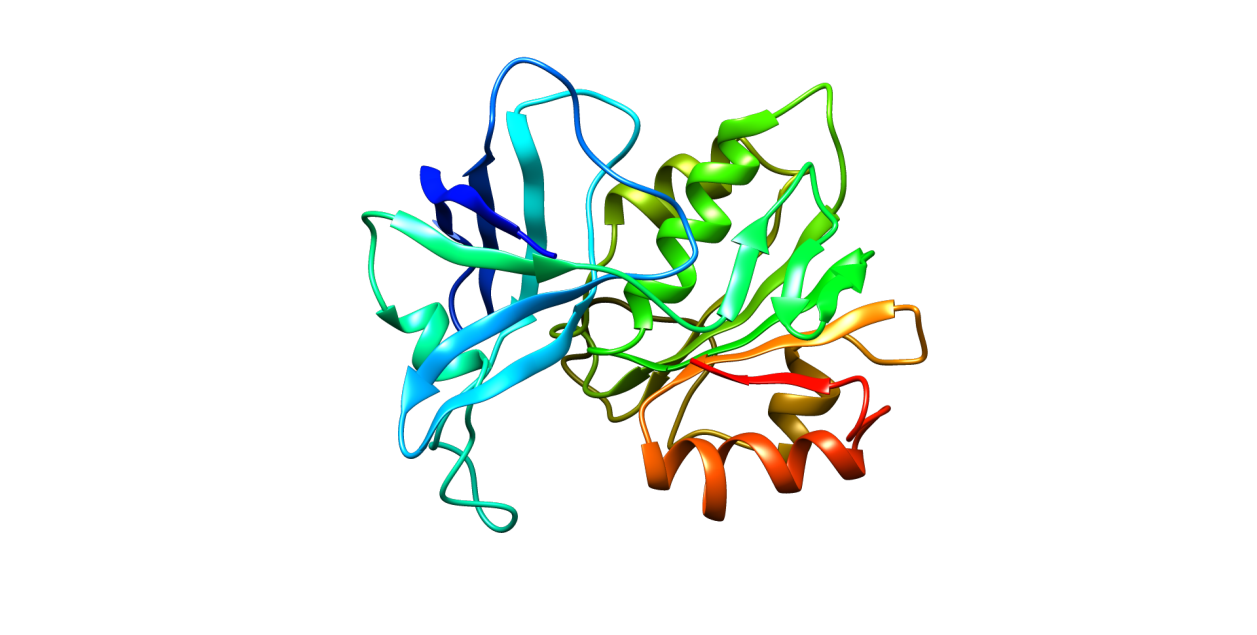

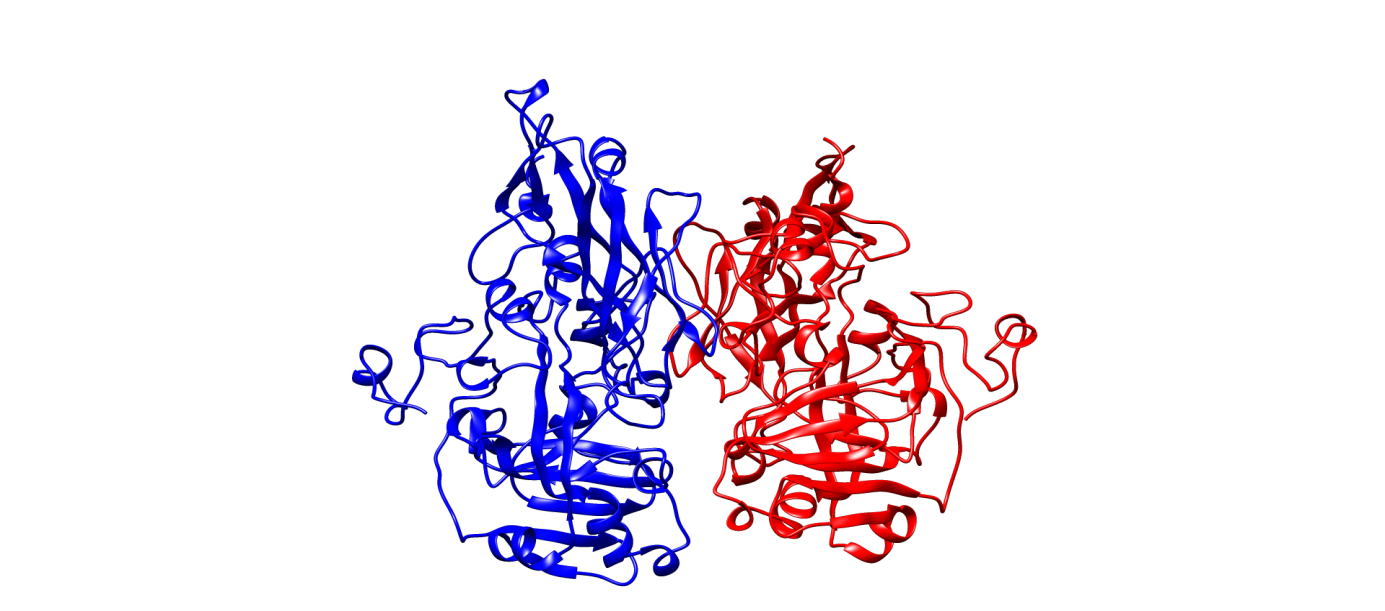

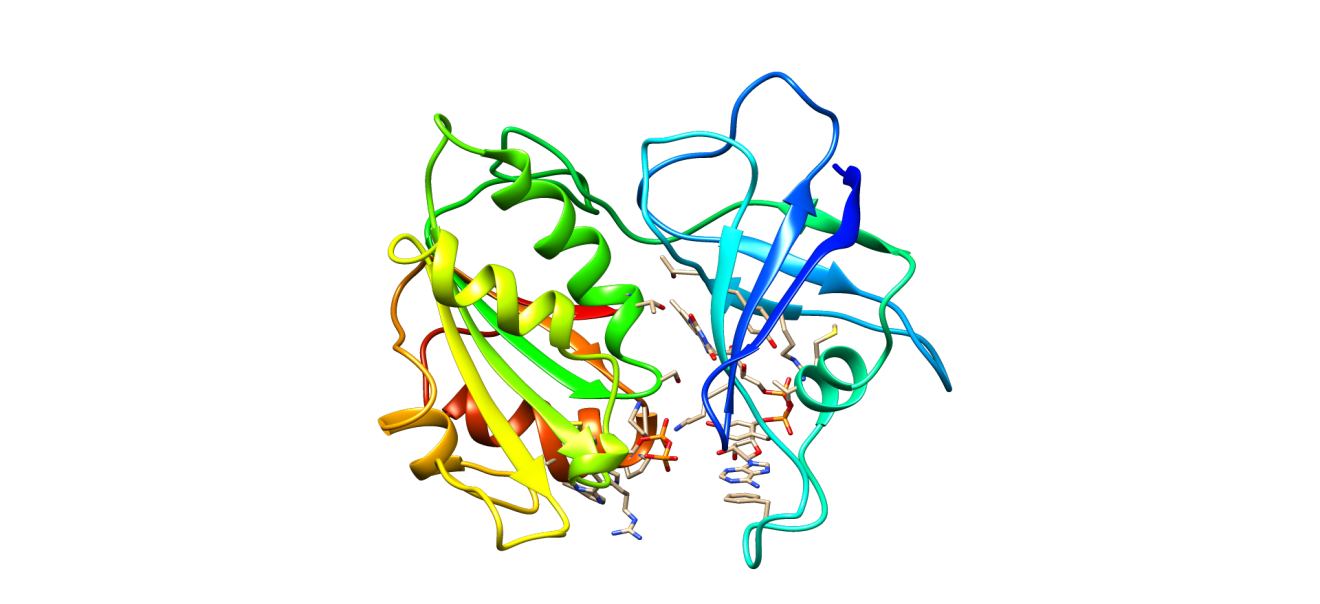

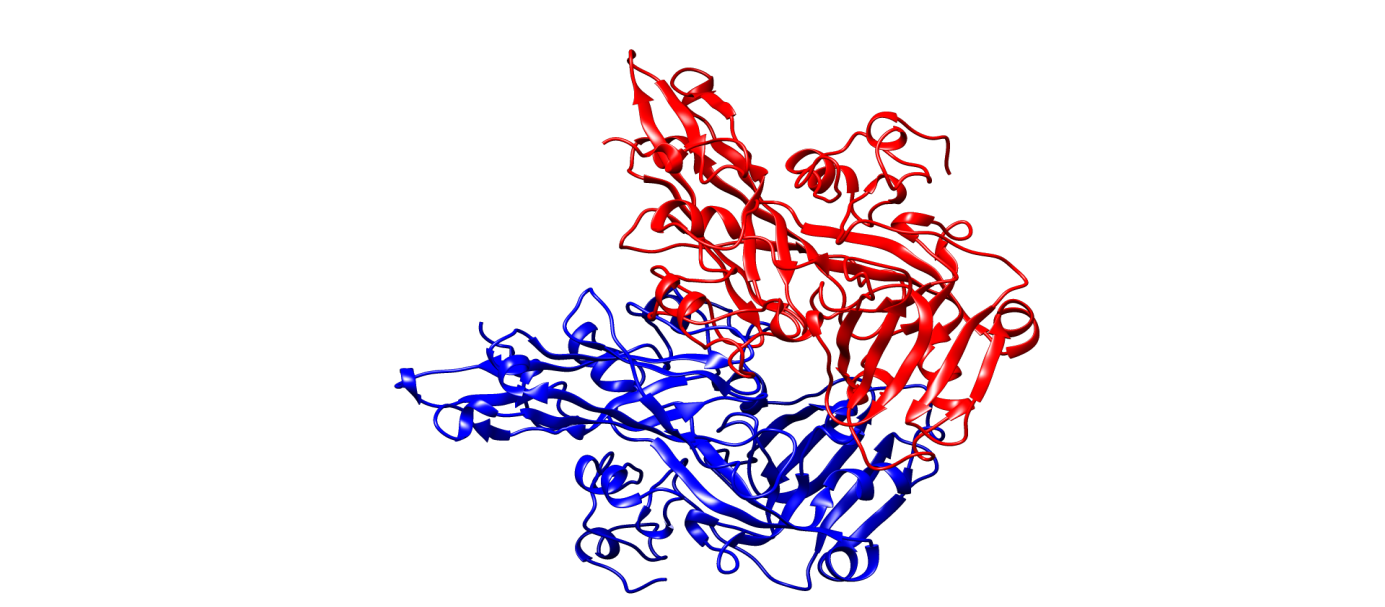

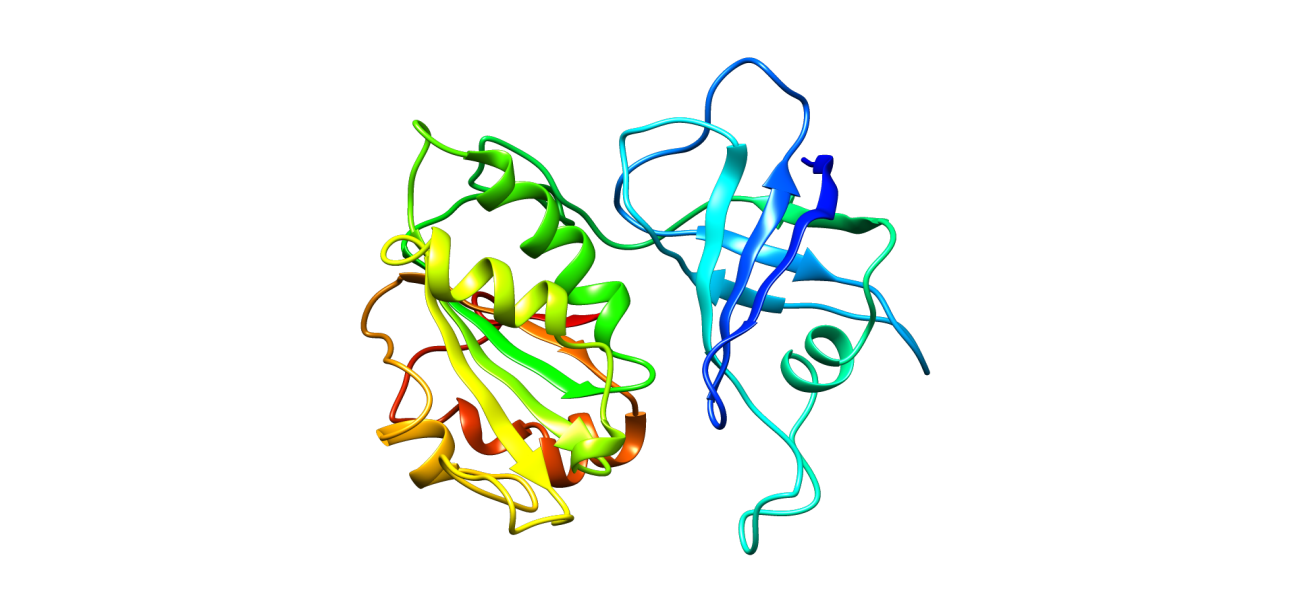

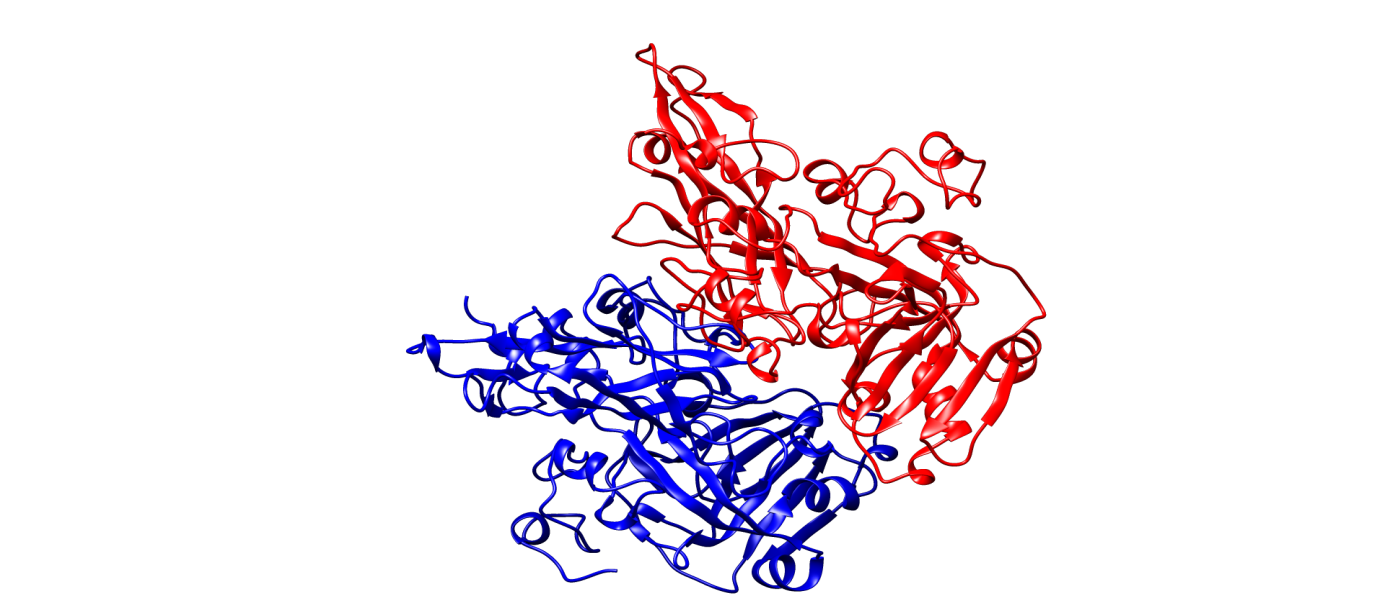

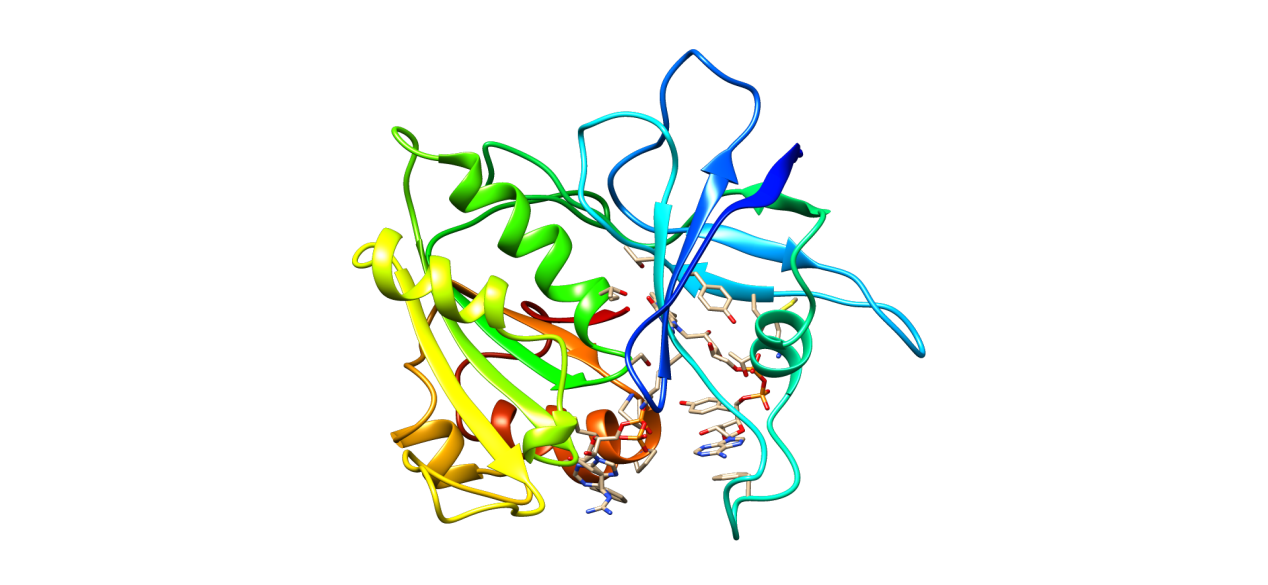

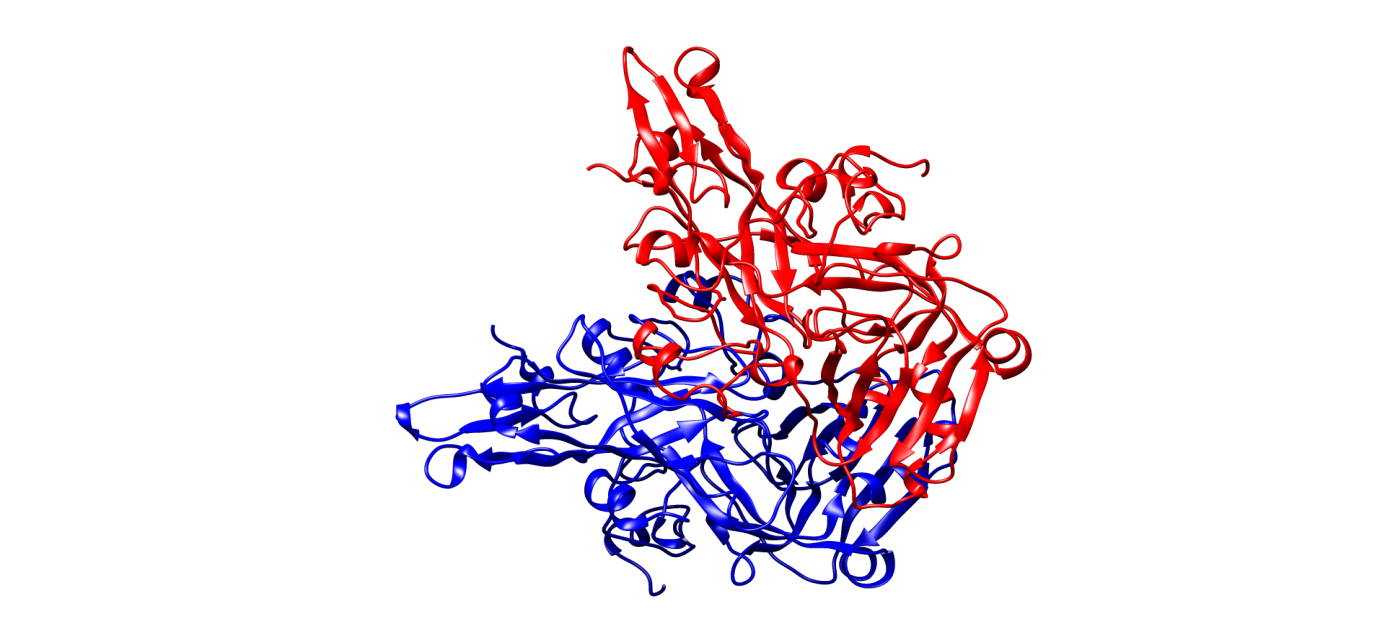

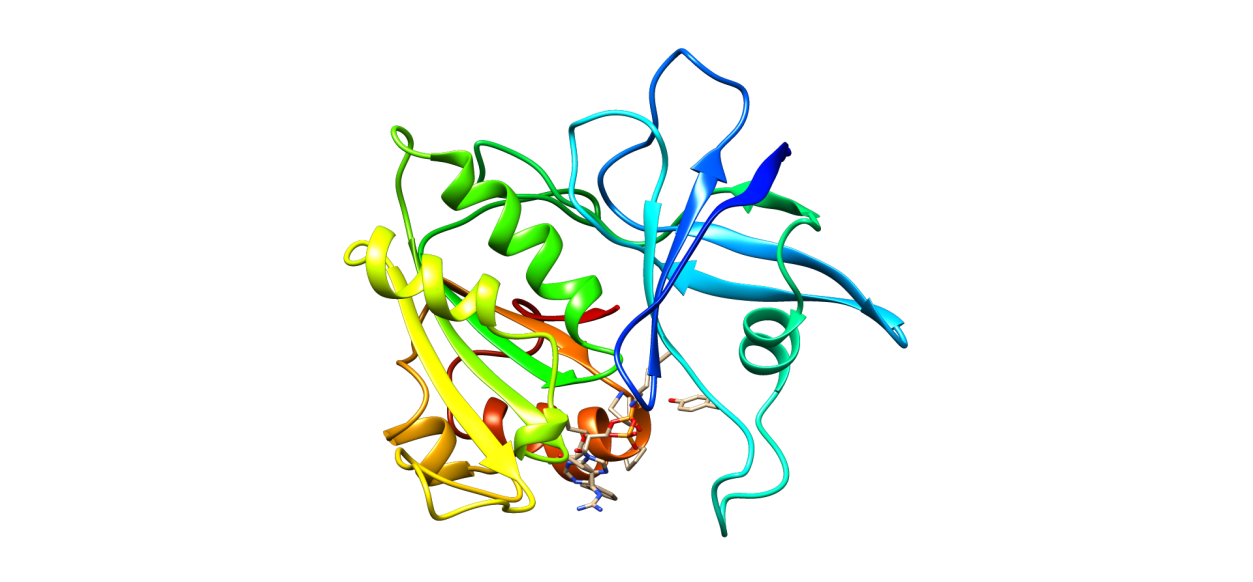

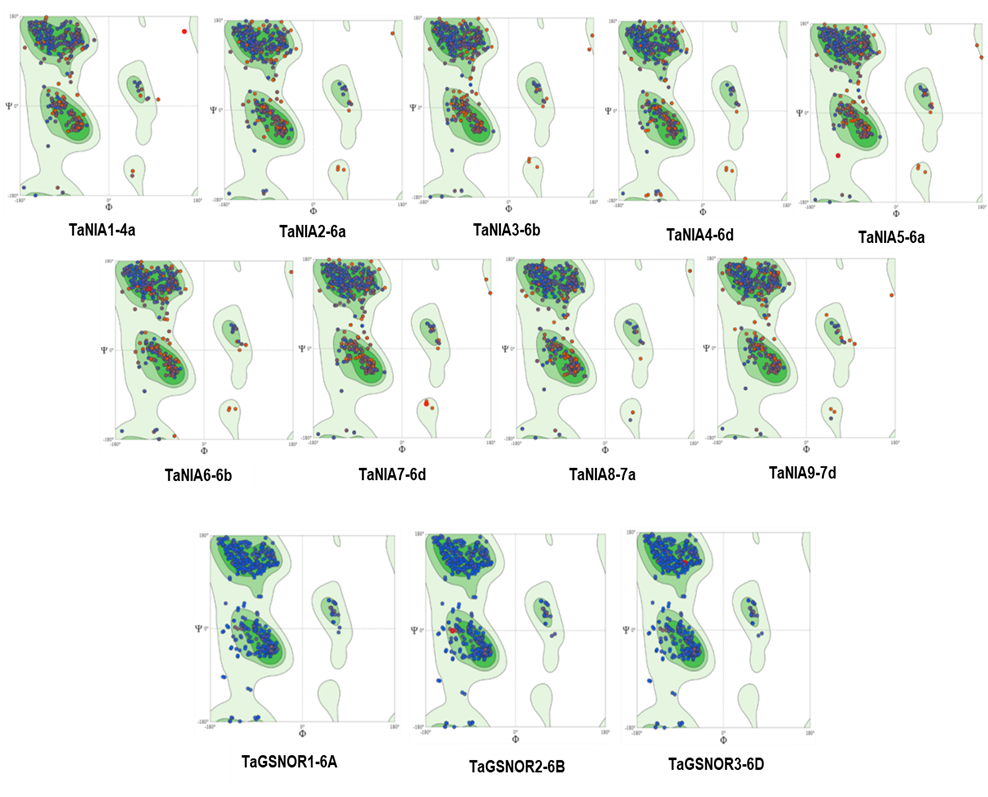


**Supplementary Figure 5** Ramachandran plot for TaNIA and TaGSNOR proteins. Images wre created using Swiss-Model server (<https://swissmodel.expasy.org/>)


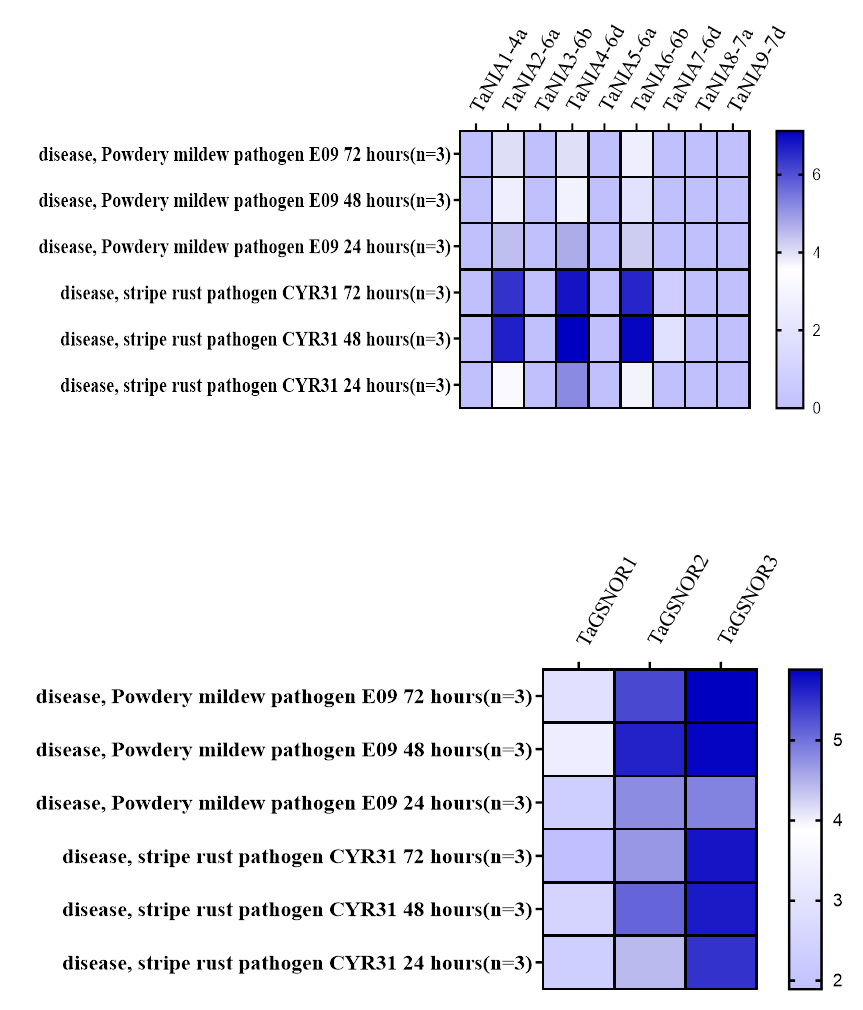


**Supplementary Figure 6** *Insilco* expression analysis of *NIA (a) and GSNOR (b)* genes in response to biotic stress perturbations showing significant expression changes at P value ≤ 0.05 using exVIP database. Images were created using exVIP (<http://www.wheat-expression.com/>).
